# Supplementary material for: Exploring thematic structure and predicted functionality of 16S rRNA amplicon data
Source: PLoS One. 2019 Dec 11;14(12):e0219235. doi: 10.1371/journal.pone.0219235 (PMC6905537; doi:10.1371/journal.pone.0219235)
Supplement: S1 Appendix — (DOCX) [file pone.0219235.s001.docx]

**S2 APPENDIX**

**RESULTS**

**Thematic Structure of IBD-Associated Microbiota (Gevers)**

**Concentration of high probability OTUs across topics begins to plateau at 75 topics.** We fit STM to the raw OTU abundance data and aimed to uncover how specific OTUs concentrate within topics as a function of topic number K (15, 25, 50, 75, 100, 150). We measured concentration using Shannon entropy. We define high quality topics as topics that place high probability on only a few OTUs; thus, high quality topics will have low entropy. A topic that is characterized by a small subset of OTUs is (1) more interpretable as a subcommunity and (2) contains more easily detectable associations with host features of interest.

For each K, we calculated the Shannon entropy for each topic, showing decreased entropy with increased topic number. This was supported by a one-way ANOVA (p<0.0001, F_5,409_=8.327) and post-hoc pairwise comparisons testing using Tukey HSD (α=0.05) (S1 Fig). Among pairwise combinations, we found that models with 75 or more topics did not have significantly different Shannon entropies, leading us to focus our attention on topic models with at most 75 topics.

**Topic modeling feasibility and generalizability**: **false negatives and rare taxa.** The largest discrepancy in classification performance between OTUs and topics was the proportion of true negatives out of total negative classifications (negative predictive value). The OTU model correctly identified CD- subjects only half the time (0.517), whereas the worst performing topic model (K=15) performed slightly better (0.526). Topic model performance improved as the total number of topics increased above 25: 0.655 (K25), 0.559 (K50), 0.577 (K75), 0.682 (K100), and 0.643 (K150) (S1 Table).

The substantially larger proportion of false negatives when using OTU relative abundances as predictive features was likely due to its reliance on few, relatively rare taxa. Topics, on the other hand, are less reliant on rare taxa, because dimensionality reduction generates less sparse predictive features. This is important because emphasizing rare taxa would hinder generalizability since the rarer the taxon, the less likely that taxon is shared across samples and the higher the likelihood that the rare taxon is absent purely due to sampling. The reliance on rare OTUs when using relative abundances can be illustrated with OTU 319708 (Clostridiaceae family), whose random forest importance score indicated that it was the fourth most important predictor for distinguishing CD- from CD+. It was more than twice as common in CD- training samples, and more than 10% of correctly classified CD- samples contained this feature. Approximately 10% of misclassified CD+ samples contained this OTU, and some of these samples contained it at a greater proportion than other samples in the training set. A similar scenario can be seen for OTU 186723 (Ruminococcaceae family), which received the highest random forest importance score for classifying CD. It was most common in CD+ samples; hence, its absence in CD+ samples resulted in false negatives.

Applying more stringent filtering or performing feature subset selection to the OTU table [1,2] (*e.g.,* lasso) prior to classification could mitigate issues stemming from emphasizing rare taxa, and may lead to better generalizability when using OTU relative abundances. However, information is lost when OTUs are removed, and the configuration of rare taxa may, in fact, be informative [3]. Feature extraction methods (*e.g.,* topic models, principal component analysis) avoid such information loss.

**Correlation Between topics and Phenotype.** We tried to discern if the subset of CD+ samples were positively associated with PCDAI. With CD+ samples, we performed reduced major axis regression between PCDAI and the relative abundances of the CD+ associated OTU profile (centered-log-ratio transformed [4]). We found a significant positive relationship (β=0.057, p=0.01, 100 permutations), albeit explanatory for only a small portion of the variation (R^2^=8.64%), suggesting that presence of this particular OTU profile may be weakly indicative of severe cases of CD (S2 Fig).

We validated how well the high-ranking-topics captured distinct profiles found in the QIIME-generated OTU relative abundance data by identifying the top-10 highest frequency OTUs in each high-ranking-topic and then comparing their prevalence in the original data (centered-log-ratio transformed). Compared to CD- samples, CD+ samples contained a disproportionate number of high-frequency OTUs from CD+ associated high-ranking-topics (S3 Fig).

Thus, the topic model approach reduced an abundance table of 1500 OTUs into 8 topics that were associated with CD status. Each topic has a distinct taxonomic configuration, and the configuration of taxa composing the CD+ associated topics were weakly correlated with PCDAI. Together, these results suggest that meaningful taxa-sample relationships can be uncovered after dimensionality reduction via the topic model.

**CD diagnosis was associated with distinct thematic profiles and hence distinct subcommunity taxonomic structure.** High-ranking-topics from the K25 model were identified as described above. Within these topics, we performed hierarchical clustering and detected multiple clusters of OTUs that disproportionately dominated topics associated with CD+. T2 contained a cluster dominated by *Enterobacteriaceae* taxa, whereas T12’s cluster contained a mixture of *Fusobacteria* and *Enterobacteriaceae*. The T15 cluster contained *Haemophilus* spp., *Neisseria*, *Fusobacteria*, and *Streptococcus*, all of which were noted as having a positive correlation with CD+ subjects in Gevers *et al.*, as well as *Aggregatibacter*, a genus reportedly associated with colorectal cancer [5].

Given that T15 contains a cluster of bacteria known for their association with bowel inflammation and this topic occurs disproportionately in subjects with greater disease severity, we asked whether the specific OTUs in T15 correlated with PCDAI. We performed negative binomial regression using abundances from the QIIME-generated abundance table (S4 Fig) and identified significant positive trends with increasing PCDAI for *Aggregatibacter* (p<0.0001, β=0.089, Z=5.285), *Erwinia* (p=0.0004, β=0.103, Z=4.116), *Fusobacterium* (p=0.0001, β=0.081, Z=6.354), and *Haemophilus* (p=0.0484, β=0.0264, Z=2.847).

The high-ranking topics for CD- (T11, T25, T13, T19), on the other hand, were dominated by taxa belonging to *Lachnospiraceae*, *Roseburia*, *Ruminococcus*, *Blautia*, *Bacteroidetes*, and *Coprococcus*, all of which were noted by Gevers *et al.* as being negatively associated with CD. In addition to these taxa, *Akkermania*, *Dialister*, and *Dorea* contributed to these topics, which is consistent with the findings of Lewis *et al.* which found a reduction of these taxa in CD+ subjects [6].

**Comparison to DeSeq2.** Pathways with the largest log-fold change (LFC) associated with CD+ samples included degradation pathways (caprolactam, LFC=0.542; fluorobenzoate, 0.532; geraniol, 0.371; and toluene degradation, 0.371), alphalinolenic acid metabolism (0.641), and electron transfer carriers (0.635).

Interestingly, the degradation pathways associated with CD+ also demonstrated strong topic-pathway interactions; however, they associated most strongly with T1, a topic unrelated to CD presence. Predicted electron transfer carrier genes were identified by both approaches, but the topic model approach isolated the effect to T12, which was also dominated by bacteria that are enriched for functions linked to secretion systems, LPS biosynthesis, and motility.

The DESeq2 approach also found fewer categories associated with CD- that had large LFC. For example, only one pathway category had a LFC less than -0.4, whereas there were 8 larger than 0.4 (indicating pathway enrichment in CD- and CD+ samples, respectively). The categories with the largest LFCs relative to CD- included germination (LFC=-0.450) and sporulation (-0.346). Similarly, the topic model identified 10 topics with functional profiles significantly enriched or depleted in sporulation genes, three of which were associated with CD- samples. Multiple topics demonstrated an inverse relationship between sporulation and LPS genes, such that topics that contained taxa enriched in one were depleted in the other.

**Within-topic co-occurrence profiles were consistent with SPIEC-EASI.** We compared topics to the correlations obtained via a network approach. The edges in the SPIEC-EASI network for the clusters of high probability OTUs in our high-ranking topics are shown in S5 Fig. For each of these topic clusters, the majority of taxa were connected by a non-zero edge (S2 Table). Of the 11 taxa in the T15 cluster, 8 had first-order connections (direct connections to other taxa within the cluster, OTU_c_-OTU_c’_), whereas 9 had second-order connections (indirect connections to other taxa within the cluster via an intermediate OTU not present in the cluster, OTU_c_-OTU_nc_-OTU_c’_). The two OTUs connected by the largest edge weight, *H. parainfluenzae* and *Haemophilus spp.*, had the highest frequencies in T15, 0.320 and 0.245, respectively. Of topics T15, T12, T2, T19, T13, and T25, none had more than one OTU with zero connections or fewer than 75% of taxa joined by first-order connections. The taxa that lacked within-cluster connections generally had low topic frequencies, with one exception, *Catenibacterium spp.* in T19. Taken together, this reaffirms that the within-topic co-occurrence profiles are consistent with alternative approaches.

**Effect of Normalization on Topic Configurations (simulation 1)**

**For small sample sizes, topic models fit with unnormalized abundances resulted in superior correspondence between simulated subcommunities and fitted topics.** We generated multiple synthetic taxonomic abundance tables with balanced and unbalanced library sizes. Unbalanced library sizes for each sample were generated by randomly sampling a new library size from a discrete uniform distribution that ranged between 100 and the original balanced library size (1000, same for all samples). We then resampled each sample from the balanced abundance table to their new library size.

Each synthetic taxonomic abundance table (*i.e.,* set of simulated samples) contained predefined subcommunities. The frequency with which these subcommunities occurred in a given sample was influenced by a binary covariate (sample class). We evaluated the ability of a topic model to recover these subcommunities as topics and assessed the influence of different normalization approaches. We considered a model satisfactory if the Kullback–Leibler divergences (KLD) between its K topics and their corresponding subcommunities were small (see methods). KLD measures the discrepancy between two probability distributions, where a value of zero indicates that the two distributions behave indistinguishably.

We found that, for small sample sizes (N=100), there was superior correspondence (smaller KLD) between subcommunities and topics when we used unnormalized abundances as opposed to rarefied or DESeq2-normalized abundances. To quantify the impact normalization strategy had on KLD, we performed multiple linear regression, with KLD as the dependent variable. The parameters we varied across simulations were used as independent variables: number of taxa in a subcommunity, number of total taxa, number of samples, number of samples containing the subcommunity, subcommunity effect size, the proportion of nonzero abundances across samples for a subcommunity (sparsity), number of topics, and normalization method (balanced library size; unbalanced library size, no normalization; unbalanced library size, rarefied; unbalanced library size, DESeq2-normalized). We compared the results relative to balanced library size samples (set as the reference level), which can be considered the ideal sequencing scenario.

The regression results indicated that KLD was roughly twice as large for rarefied (β_coef_=0.397 (the coefficient for the indicator for rarefied library size compared to balanced library size), SE=0.0309, p<0.0001) and DESeq2-normalized (β_coef_=0.369, SE=0.0309, p<0.0001) data compared to unnormalized data (β_coef_=0.189, SE=0.0309, p < 0.0001, R^2^=0.736). This indicated that both rarefying and DESeq2 normalization negatively affected the ability of topics to recover predefined subcommunities. For DESeq2 normalization, this trend persisted for all subcommunity effect sizes (mean abundance of a subcommunity) but dampened as abundances became less sparse (S6 Fig).

With a larger sample size (N=500), as the synthetic abundance data became less sparse, the differences between normalization methods became less pronounced. This was largely due to the effect rarefying and DESeq2 normalization had on rare taxa. Rare taxa are more likely in sparse datasets with few samples. Rarefying down-samples taxa abundances; thus, rare taxa are less likely to be resampled. DESeq2 normalization, on the other hand, can result in negative values for rare taxa. These values must be set to zero prior to fitting a topic model. Thus, in both cases, relatively rare taxa have little to no influence on topic estimation, which likely impacted the ability of topics to accurately map to the predefined subcommunities.

We also estimated the likelihood that the different normalization methods would produce multiple, largely overlapping topics (topics with low KLD from more than one subcommunity) using overdispersed binomial regression. The number of these redundant mappings was set as the dependent variable, with the same covariates listed above as the independent variables. We found no relationship between redundancy and either rarefied (β_coef_=0.022, SE=0.015, p=0.145) or unnormalized (β_coef_=0.019, SE=0.015, p=0.221) abundances, but found a positive association with DESeq2-normalized abundances (β_coef_=0.038, SE=0.015, p=0.013). This suggests that the dampening effect on rare taxa was greater for DESeq2 normalization than rarefying, resulting in inferior topic mappings for DESeq2.

**DESeq2 normalization is more conservative at detecting binary topic-sample-effects.** We next assessed the effect of normalization on statistical power (the likelihood of detecting relationships between sample covariates (*e.g.*, disease status) and the frequency of a topic in a sample). We term the relationship between sample covariate and topic frequency in a sample as a topic-sample-effect. For example, topics associated with the presence of a particular disease should be more prevalent (higher frequency) in diseased samples. The number of detectable topic-sample-effects increased as subcommunity effect size increased and subcommunities became less sparse (S7 Fig).

We again turned to overdispersed binomial regression with the total number of detected topic-sample-effects set as the dependent variables (the independent variables were the same as above). DESeq2 normalization was the most conservative technique, frequently resulting in fewer detectable topic-sample-effects (β=-0.169, SE=0.034, p<0.0001). Performing no normalization had seemingly no impact on the ability to detect topic-sample-effects (β_coef_=-0.059, SE=0.034, p=0.077). Increasing the total number of topics from 15 to 50 drastically diminished power for all normalization procedures (β_coef_=--0.593, SE=0.024, p<0.0001), particularly when the sample size was small (N=100). Of note, for models with 50 topics, balanced library sizes resulted in at most one detectable effect for all parameterizations except when sample size was increased from 100 to 500, which resulted in a considerable increase in the number of detectable effects, with DESeq2 again behaving most conservatively.

Together, these results suggest that correcting for library size imbalance via DESeq2 normalization or rarefying is unnecessary when fitting a topic model to taxonomic abundances and possibly detrimental when sample size is small. DESeq2 normalization decreased power for detecting topic-sample-effects and slightly increased the frequency of redundant topic mappings. When sample size is small, rarefying and DESeq2 normalization negatively affected the ability of the topic model to recover subcommunities compared to directly using unnormalized abundances.

The performance of rarefying would likely improve by using a larger common depth for down-sampling as shown in [7]; however, many datasets are often shallowly sequenced, necessitating the use of a small common depth [8–12]. The poor performance of DESeq2 normalization, on the other hand, is likely due to rare taxa receiving negative normalized values, which must be set to zero prior to fitting the topic model. This dampens the contribution of rare species on inferring topic structure. A seemingly obvious adjustment would involve shifting the normalized values by a constant, but this is incorrect because the normalized values are logarithmically transformed [13]. An alternative approach worth exploring could involve a centered-log-ratio transformation using a Box-Cox transformation as opposed to a log transformation. While negative values would still occur, with the appropriate parameters, there may be fewer, resulting in greater contribution by rare species for topic estimation. However, like DESeq2, this approach would require one to calculate the geometric mean across samples, which tend to be sparse (many abundances of zero). Thus, there is still need to identify an improved strategy for handling zeros when calculating the geometric mean, since using pseudocounts by simply adding a constant has been shown to yield spurious results [13,14].

**Event Detection (simulation 2)**

**Clusters of correlated topics successfully captured short-lived patterns.** As in simulation 1, we evaluated the ability of a topic model to capture, as topics, subcommunity structure. However, for this simulation, the mean abundance of a subcommunity varied over time (see methods). The patterns in which they varied were influenced by combinations of three classes of temporal patterns: short-lived patterns (pulses), long-term changes (steps, *i.e.,* sudden increase to a new mean abundance) and steps with decay (gradual increase to a new mean abundance), and oscillatory patterns (oscillations). We generated 12 synthetic taxonomic abundance tables, each with their own set of subcommunities that varied by their own predefined temporal pattern (trajectory sets, ts) (S8 Fig top, S9 Fig top). Trajectory set 1 contains 3 subcommunities with pulse patterns at different time points. Trajectory sets 2 and 3 also contain pulses, in addition to subcommunities that gradually increase in abundance over time. Trajectory sets 7-10 contain oscillating subcommunities. Trajectory set 7 contains a single subcommunity. Trajectory set 8-10 each contain two subcommunities. Trajectory set 8’s are sinusoidal and out of phase, whereas trajectory set 10’s are square waves. Trajectory set 9 contains a pulse and oscillating subcommunity that gradually increases in amplitude over time. Lastly, trajectory sets 11 and 12 contain pulse subcommunities, but the taxa that constitute these subcommunities overlap considerably.

To recover the subcommunities as topics, we identified topics that co-occurred across samples. As recommended by Roberts *et al.* [15], we built a sparse correlation network using the topic-to-topic covariance matrix estimated by the topic model. We used the network to identify “topic clusters” of correlated topics. Then, from the posterior distribution we obtained frequencies in which topics occurred in samples and taxa occurred in topics. We used these frequencies to assess (1) the frequency in which topic clusters were sampled given time and (2) the taxonomic composition of these clusters. We applied four quality scores to measure the correspondence between subcommunities and topic clusters: F1, purity, cluster Root Mean Squared Error (RMSE), and taxa RMSE (S3 Table).

S8 Fig shows the scores for the best topic clusters (in terms of F1 score) for 9 of the 12 trajectory sets (ts 1-12) and subcommunities (sc 1-5). The model effectively recovered pulses (ts 1; ts 3, sc 1; ts 5, sc 2-4; ts 9, sc 1; ts 11; ts 12). Nearly all the 10 best F1, cluster RMSE, and taxa RMSE scores resulted from subcommunities displaying pulse patterns (S9 Fig). In addition, the topic clusters mapping to these subcommunities did well at capturing the subcommunity’s taxa; the top-10 clusters in terms of F1 score had purity scores ranging from 0.421 (error = +/- 0.060) to 0.628 (+/- 0.063), suggesting that roughly half of all taxa populating these topic clusters belonged to these simulated (and hence known) subcommunities. To further quantify the purity of the topic clusters we identified which topic clusters mapped to the 10 subcommunities displaying pulse pattern. Then, from the posterior distribution, we calculated the sampling frequency of all taxa in these clusters, resulting in posterior sampling frequencies for 250 types of taxa. When we ranked the frequency with which each taxon was sampled, no subcommunity member ranked lower than 23rd (out of 250), indicating that our approach performed well at identifying the taxa that constitute pulse changes among microbiota.

We next compared the top performing topic clusters (based on cluster RMSE) to the top performing clusters identified via HC, an approach commonly used to learn the similarity of taxa based on their abundances across samples (*i.e.*, their co-occurrence). The topic model resulted in topic-subcommunity mappings that had slightly worse cluster RMSE (more error) compared to HC clusters (S8 Fig). Across the 12 tables, RMSE was lower roughly 70% of the time for the HC clusters compared to the topic clusters (Fig S9). There was also a significant difference in mean cluster RMSE between the two approaches (paired t-test, t=2.36, df=32, p=0.024), but not mean purity (t=-0.272, df=32, p=0.787). While these results suggest that the HC approach had lower error and hence outperformed the topic model approach, note that we based the number of HC clusters (30) on our knowledge of how many taxa formed a subcommunity (8) and how many taxa there were in total (250). Experimental datasets would lack this luxury. Moreover, because the choice of 30 clusters resulted in near-optimal HC cluster size, the resulting RMSE would be at a minimum if the taxa making up the cluster well-approximated the true subcommunity taxonomic composition. Thus, the HC RMSE that we show is a lower-bound best-case scenario.

**Clusters of correlated topics recovered the periodic signals and outperformed HC in terms of purity.** For the topic model approach, oscillations (ts 7, 8, 10; ts 9, sc 2) posed a difficult task because multiple topics often captured different segments of a long-term temporal pattern, making reconstruction of the pattern difficult. Segmentation of a pattern was likely increased by the topic model’s sparsity-promoting (regularizing) prior distributions, as well as a large number of topics. For instance, the estimated taxa frequencies across topics is sparse, such that, for a particular topic, the majority of taxa have low probability of being present. This also applies to the estimated topic frequencies across samples; few topics represent a particular sample. Thus, if sample progression represents time (as in our case) and the configuration of taxa evolves over time (such as the patterns seen in our synthetic subcommunities), then as the configuration of taxa changes, different small sets of topics emerge with high frequency, which seemingly presents as a sequential set of pulses (individual, high frequency topics) (10C Fig). This concern of a sustained temporal pattern segmenting into multiple topics (pulses) inspired us to utilize topics-to-topic correlation (S10B Fig) to construct topic clusters from correlated sets of topics. We posit that these topic clusters should reconstruct long-terms patterns (10D Fig) from short-term segments (topics) (10D Fig) if the topic correlation structure accurately represents topics that co-occur over time.

To further investigate the performance of the topic models with periodic signals, we compared subcommunities with long-term, oscillating temporal patterns (ts 7, 8, 10; ts 9, sc 2) to their corresponding reconstructions using the clusters identified via HC and the topic model approach. Here, we are focused specifically on whether the HC or topic clusters identify the correct set of taxa that correspond to the subcommunity of interest. For a given trajectory (subcommunity) from a trajectory set (*e.g.,* ts 7, sc 1), we generated HC clusters and topic clusters as described previously. For an HC cluster, which is composed of a distinct set of taxa, we subset only those taxa from its synthetic abundance table and compare that (HC) subset’s total (summed) abundance over time to the abundance over time of the true subcommunity. For a topic cluster, we sample, from the posterior distribution, taxa given time, then identify high frequency taxa (see methods). With this subset, as before, we acquire its total (summed) abundance over time from its synthetic abundance table to compare it to the abundance over time of the true subcommunity.

S11A Fig shows the true subcommunity abundance over time (black), as well as the reconstructed abundances over time from the best performing HC and topic clusters (green and red, respectively). Generally, these clusters managed to reconstruct the oscillations of each subcommunity. When we inspect the mean absolute residual error (MAE) (S11B Fig), we can see that the HC cluster had most difficulty reconstructing subcommunity 2 from trajectory set 9 and subcommunity 1 from table 10. The topic clusters, on the other hand, had most difficulty reconstructing the subcommunities from trajectory set 8, but showed better performance relative to HC for all other trajectory sets. This suggests that forming topic clusters from correlated sets of topics effectively identifies the correct subset of taxa in a subcommunity that demonstrates long-term oscillatory behavior.

When we shift our focus to F1 score and RMSE, we can see that the performance of topic clusters in capturing long-term patterns (trajectory sets 7, 8, 10) was worse compared to short-term patterns (trajectory sets 1, 11, 12). Relative to long-term changes, oscillations resulted in worse F1 scores and larger cluster RMSE. For oscillatory patterns, the best performance was for subcommunity 1 in trajectory set 10, using 50 (F1=0.655 +/- 0.054) and 65 topics (F1=0.666 +/- 0.048) (S4 Table). This table is notable for two oscillating (s-waves) subcommunities that never had non-zero abundances on the same day (sample), such that for a given week, subcommunity 1 occurred only during first 4 days, whereas subcommunity 2 occurred only during the remaining 3 days. On the other hand, trajectory set 8 involved two out-of-phase oscillating (sinusoidal) subcommunities. Consistent with the results above, the topic model managed to identify the taxa that made up each of the four oscillating subcommunities. For models with 50 topics, the best cluster-subcommunity mappings had purity scores of at least 0.679, with subcommunity 1 in trajectory set 8 performing best at 0.980 +/- 0.014. This was a general trend, with the topic model approach outperforming HC in terms of purity for most (5/6) oscillating subcommunities. Moreover, the average purity for the 6 HC clusters was 0.420, with two clusters as low as 0.167 and 0.133, suggesting an inability for HC to adequately capture the taxonomic composition of oscillating subcommunities. On the other hand, mean topic cluster purity was 0.662.

**Patterns with overlapping taxa negatively affected topic purity.** Purity suffered the most for subcommunities displaying pulse patterns that overlapped in terms of taxa composition (they were composed of similar subsets of taxa) (ts 11-12). Roughly half of all clusters in trajectory sets 11 and 12 had purity scores less than 0.388. The inability of topic clusters to accurately identify the taxa found within these subcommunities was due to redundant topic cluster mappings (distinct topic clusters containing taxa from multiple subcommunities). For example, for the model with 10 topics, one cluster in table 11 mapped to subcommunities 2 and 3, which shared 4 taxa. This also indicates why trajectory sets 11 and 12 had taxa RMSE that was lower than cluster RMSE.

Compared to HC clusters, topic clusters with low cluster RMSE had higher purity roughly 63% of the time for trajectory sets 11 and 12. For the phase-shifted, oscillating subcommunities found in trajectory set 8, the topic model approach generated substantially purer clusters (0.550 and 0.634 for subcommunities 1 and 2, respectively) compared to HC (0.133 and 0.429). We speculate that for overlapping subcommunities composed of more taxa, the topic clusters would further outperform the HC approach. Moreover, in experimental scenarios where there is little information regarding the true subcommunity size, HC cluster purity would decrease. The topic model approach, on the other hand, is more robust to circumstances where the cluster size is unknown, since combinations of topics that individually underestimate (segment) the true subcommunity size can together better reconstruct the complete subcommunity configuration. These combinations of topics can be inferred by leveraging topic-to-topic correlation.

**Thematic Structure of Temporally Varying Microbiota (David)**

**Measuring time series event effect size.** We quantified the community-wide shift of taxonomic abundances with canonical correspondence analysis (CCA) and PERMANOVA (via the adonis function in the R package vegan [16]). For each synthetic time-series from simulation 2, we used binary indicators for each intervention as covariates. We then calculated the proportion of constrained inertia and R^2^ for CCA and PERMANOVA, respectively. We repeated this approach using the David et al. dataset, using two covariates, where covariate 1 was 1 for days 1-150 and 0 otherwise, and covariate 2 was 1 for days 160-189 and 0 otherwise.

**Time series reconstruction via PCA.** We explored PCA as a means to reconstruct the time-series patterns. S12 Fig shows the reconstructed patterns for each of the 12 trajectory sets, which suggests that PCA could capture the underlying signal. However, the reconstructed pattern is often a combination of patterns from more than one SC, rendering the result impure and hence misleading. In addition, because we lacked a straightforward approach to recover the underlying taxa that compose a particular signal, we had no way of calculated RMSE to compare to the other approaches. This limitation alone suggests that using a PCA to capture dynamic SC behavior is limited.

**HC resulted in a wider estimate for the length of the illness profile for David *et al*.** With HC, we created 6 clusters based the three profiles reported in David *et al*. (S13 Fig.) Note that we did explore other parameterizations, which yielded similar cluster configurations with respect to both time and taxonomic composition (S14-15 Figs.). Since we used a priori knowledge, identification of these clusters can therefore be considered a best-case-scenario. Three clusters (2, 3, 6) corresponded to the days in which subject B presented with food poisoning. Clusters 5 and 6 were comprised of 355 and 298 taxa, respectively, and, in the raw relative abundance table, both peaked on roughly days 151 to 157. However, the taxa in these clusters during this span were low-frequency taxa; all had mean relative abundance less than 0.0002. In cluster 5, the taxa with largest mean relative abundance included *H. parainfluenzae*, *Leuconostocaceae spp., Dialister spp.,* and *Enterobacteriaceae spp.*, whereas cluster 6 included *Klebsiella spp., Closridiaceae spp.* and *Enterobacteriaceae spp*. Cluster 2, on the other hand, spanned days 151 to 169, and contained taxa considerably larger in terms of mean relative abundance: *Bacteroides spp.* (mean relative abundance=0.192)*, Enterbacteriaceae spp.* (0.034)*,* and *H. parainfluenzae* (0.013) composed this cluster. Together, these three clusters likely correspond to profile 2 identified by David et al. (days 145 to 162) and clusters 5 and 6 (151 to 157) specifically correspond to the time of illness estimated by both the topic model (153 to 158) and David et al. (151 to 159). However, unlike the topic model approach, these clusters consist of substantially more taxa and hence are inundated with more noise.

Cluster 4 contained 360 taxa and corresponded well to the pre-illness period, spanning days 1 to 150. During this span, large mean relative abundance taxa that associated with cluster 4 included *Bacteroides spp* (0.156)*, Lachnospiraceae spp.* (0.078)*,* and *Faecalibacterium praunitzii* (0.050). This set of taxa was similar to the taxa identified in the topic model’s profile 1. The post-illness period (profile 3) was captured by clusters 1 and 3, but these clusters failed to completely separate profile 2 from profile 3; they spanned days 151 to 318. They were composed of taxa similar to cluster 4, but with a substantial contribution from the family Ruminococcus, a change seen in profile 3 for the STM. The top mean relative abundance taxa in clusters 2 and 3 were *Ruminococcus spp.* (0.132)*, F. prausnitzii* (0.112)*, Bacteroides spp.* (0.086)*,* and *Lachnospiraceae spp* (0.025).

These results suggest that the profiles identified via the topic model approach are similar to those obtained via HC. However, the sparsity inducing priors in STM ease interpretation since the profiles are less contaminated with unimportant taxa. The smallest cluster obtained with HC contained 121 taxa (cluster 1). Without prior knowledge to suggest where the breaks between profiles may occur, identifying meaningful abundance profiles (during the tree cutting stage or the analysis stage) may be increasingly difficult. Also, the topic model approach identified topics that likely represented the initial presentation of the illness (day 153, topic 9) and a sequence of topics that shows a gradual evolution of the abundance profile (topic cluster 2). The clusters associated with disease obtained via HC unsuccessfully separated the shift from profile 2 to 3 and, moreover, where unable to demonstrate how the profiles evolved over time.

**Shifts in the taxonomic abundance profiles for the synthetic time-series from simulation 2 were similar to the shifts observed in the David *et al.* data.** Given how clear the delineations between shifts in gut profiles were, we attempted to quantify the degree in which the David *et al*. profiles changed before and after the subject’s bout with food poisoning. Doing so enabled us to compare the signal seen in David *et al.* to our synthetic datasets from simulation 2. We used proportion of inertia (via CCA) and R^2^ (via PERMANOVA) for a given signal as our measure of effect size. The results are shown in S5 Table, which indicate that the David *et al.* signals represent slightly less total variation compared to the synthetic datasets, with the periodic datasets 7 and 8 being most similar.

**METHODS**

**Assessing Concentration of OTUs as a Function of Topic Number**

For each STM (K ∈ 15, 25, 50, 75, 100, 150, 250), Shannon entropy was calculated for each topic in the topics-over-OTUs distribution. To compare mean entropy across STMs, we performed an ANOVA, followed by Tukey HSD post-hoc analysis.

**Comparing Topic Taxonomic Profiles to a Network Approach**

To further validate the clusters of high frequency taxa identified in the topics-over-OTUs distribution, we compared our results to those generated from an OTU-OTU association network on the copy number normalized OTU relative abundances using SPIEC-EASI’s neighborhood selection method (lambda.min.ratio=.01, nlambda=20) [32].

**Simulation 1**

S16 Fig shows our approach for simulation 1. We will refer to the figure sub-blocks 1-10 throughout this section. (1a) To create the synthetic absolute abundance table (balanced table), we first generated a background distribution of size $M^{\left( \mathrm{samples} \right)}\times N^{(taxa)}$ from a zero-inflated negative binomial distribution (ZINB) with sparsity (φ), mean (μ), and size (ψ) parameters adjusted to match distribution characteristics found in datasets such as Gevers *et al*. (*e.g.,* sparsity, variance, max, etc.) [17]. The ZINB was chosen given its ability to simulate the excessive zeros and overdispersion often encountered in 16S rRNA gene surveys [18]. The dimensions of the taxonomic profile were a function of the number of samples M ∈ {100, 500} and number of taxa N ∈ {500, 1000} in a given simulation. (2) We randomly split simulated samples into equally sized treatment and control groups. (3) We then created 15 (arbitrary total) mock subcommunities of size sc_l_ ∈ {10, 15, 30}, composed of non-overlapping taxa that were generated by resampling with replacement all nonzero values in the background distribution and then scaling these values by effect size sc_m_ ∈ {1, 2, 5, 10} and setting a proportion 1-sc_p_ (sc_p_ ∈ {0.10, 0.25, 0.5,.0.75}) of these values to zero. Of these 15 subcommunities, 5 were set to replace the taxa abundances from a proportion g_p_ ∈ {0.25, 0.50, 0.75} of treatment samples, 5 from a proportion of control samples, and 5 to replace an equal proportion from both treatment and control samples.

From the balanced table, we generated a second (unbalanced) table to investigate the effect of varying library size on model performance. (1b) Library sizes for each sample were randomly generated from a discrete uniform distribution [100, min(sample sum)] and used to resample the background distribution. (1c,d) The unbalanced table was then either rarefied to a balanced library size (N_min_=1000) (“rarefied” table) or normalized using the DESeq2 variance stabilizing transformation (“DESeq2” table) to create two relative abundance tables. Rarefying is a normalization approach where samples are down-sampled to a minimum value N_min_, and any samples falling below this value are discarded. DESeq2 [19] normalization is a variance stabilizing technique that adjusts discrete abundance data in terms of its mean-variance relationship and within-sample geometric mean.

(4) After generating the rarefied and DESeq2 tables, topic models were fit to obtain thematic representations of the four simulated abundance tables. Model performance was assessed in two ways. First, we performed linear regression for each topic, using the frequency of topic k across samples ($\theta_{\cdot,k}$) as a dependent variable and the binary indicator for treatment and control as the independent variable. We refer to the estimated regression coefficients as topic-sample-effects. For each coefficient, we calculated 95% uncertainty intervals. Intervals that do not span zero are referred to as detectable topic-sample-effects.

(5-7) Second, we calculated KLD between p(x_n_|SC_w_)_data_ and p(x_n_|SC_w_,k)_model_, resulting in a score for each topic-subcommunity pair for a given model parameterization. It should be noted that while we chose KLD, we did explore the use of other information metrics such as Jensen Shannon distance; the results were equivalent. (8-9) For a given topic model with K topics, we identified the minimum threshold $\tau$ in which there remain K KLD values less than $\tau$ (equation 1):

$$\tau^{*}=\mathrm{argmin} \left\{ \tau\geq0 | \sum_{k,w} 1\left[ \mathrm{KLD}_{k,w}<\tau\right]\geq K \right\}$$

Equation 1.

where K is the number of topics, KLD*_k,w_* is the KLD between p(x_n_|SC_w_,k)_model_ and p(x_n_|SC_w_)_data_ and 1 is the indicator function that returns 1 if KLD*_k,w_ <* $\tau$ and 0 otherwise.

We posited that an outcome with good predictive power occurs when at least K topics mapped to a subcommunity; fewer than K mapped topics guaranteed that some subcommunities were unaccounted for. These K values represent the K topics with smallest KLD to a subcommunity. (10) We summed the number of subcommunities to which each of these K topics mapped (“redundancy scores”). Topics with small KLD to multiple subcommunities (a large redundancy score) would imply an inability of the topic model to separate subcommunities and thus capture their unique co-occurrence profiles. An ideal result would be each topic mapping uniquely to a single subcommunity. We consider a many-to-one mapping acceptable, where multiple topics map to a single subcommunity, if the topics map to one and only one subcommunity.

**Assessing simulation 1 performance.** To infer the relationship between simulation parameters and threshold value, we performed multiple regression with the following scaled and centered covariates: number of taxa in a subcommunity, number of total taxa, number of total samples, proportion of samples receiving the subcommunity, subcommunity effect size, subcommunity sparsity, number of topics, and normalization method. For the normalization method factor (DESeq2, rarefied, unbalanced, balanced), we set “balanced” as the reference level (*i.e.,* the intercept). KLD threshold values were log transformed and used as the dependent variable. For redundancy score, we performed overdispersed binomial regression using the same set of covariates and setting K as the number of Bernoulli trials.

To assess the degree in which a given normalization procedure dampens topic-sample-effects, for all parameter combinations, we quantified the proportion of detectable topic-sample-effects. We then performed overdispersed binomial regression with the following scaled and centered covariates: number of taxa in a subcommunity, number of total taxa, number of total samples, proportion of samples receiving the subcommunity, subcommunity effect size, subcommunity sparsity, number of topics, an indicator value representing whether a binary covariate for treatment verses control was present in Z (where $\theta\sim LN_{K-1}(\Gamma^{T}Z_{m}^{T},\Sigma)$), and normalization method.

Quality of fit for all regression models was assessed by testing for equal variance and normality of the model residuals. Coefficients were considered statistically significant at p < 0.05.

**Simulation 2**

Synthetic abundance tables were created to assess the ability of the topic model to detect time-series patterns displayed by subsets of co-occurring taxa. Our approach was heavily influenced by the simulation detailed by Hall *et al*., who utilized Ananke to perform temporal clustering [20], but differs in the way we generated our synthetic abundance tables and our patterns. S17 Fig shows our approach for simulation 2. We will refer to the figure sub-blocks 1-3 throughout this section.

(1) We first generated 12 background distributions of 250 taxonomic features across 100 time points using the same ZINB distribution described in simulation 1. Then, we defined a subcommunity as a set of 8 (arbitrary total) taxa. (2-3) We agitated various subcommunities by multiplying the background distribution by one of 3 types of patterns: pulses (S17 Fig: I1, I2), steps (I3, I4), and oscillations (I5, I6).

A pulse P is defined as a short-term event where there is a mean shift in the background distribution for fewer than 5 time points T:

$$P_{t}^{T}=\left\{ \begin{aligned} 1, if t=T \\ 0, \mathrm{otherwise} \end{aligned} \right.$$

A step S extends from the initial pattern time point T_1_ until either end of the time-series:

$$S_{t}^{T}=\left\{ \begin{aligned} 1, if t\geq T \\ 0, \mathrm{otherwise} \end{aligned} \right.$$

Oscillation P is defined as cyclical behavior that may or may not occur for the entirety of the time-series:

$$P_{t}^{T}=\left\{ \begin{aligned} \sin\left( 2\times\pi\times f\times t+\epsilon_{1} \right)+cos(2\times\pi\times f\times t+\epsilon_{2}), if t=T \\ 0, \mathrm{otherwise} \end{aligned} \right.$$

where f is the frequency of the signal and ε are the phase shifts.

Pulses and steps may include a weight that influences the rate of decay; that is, the rate in which the subcommunity returns to its pre-pattern behavior (I2, I4). Using our set of patterns, we generated 12 synthetic time-series abundance tables. Samples were regarded as daily observations. All oscillatory patterns were fast, having a weekly period of 7 days (f=1/7). We posited that weekly periodicity is relevant to simulating gut microbiota dynamics.

For fitting the STM, we treated the time index as a covariate representing day and created a second covariate representing day-of-week (DOW). Each synthetic dataset was fit with an STM $(K\in\left\{ 10, 20, 35, 50, 65, 80, 100 \right\})$ that included a non-linear spline with 10 degrees of freedom on day and a second-degree polynomial on DOW.

**Event detection.** For a given STM parameterization, we calculated the topic-to-topic correlation network graph via Zhao and Lui [21], which is available in the R package *stm* [15] and wrapped in our package *themetagenomics* [22]. Briefly, the procedure first performs a non-paranormal transformation on the samples-over-topics distribution θ to alleviate the normality assumption. It then estimates the graph via the Meinshausen-Buhlmann method, which uses L1 regularization and is hence suitable for high-dimensional data [23]. Selection of the regularization parameter was performed via the stability approach to regularization selection. We parsed the resulting correlation network to identify cycles, linear chains, and clusters, which are defined as follows: a cycle consists exclusively of vertices of degree 2, forming a closed chain; a linear chain consists exclusively of vertices of degree 2, except at its ends, where each end may connect to a larger subnetwork; and a cluster is a set of interconnected vertices of varying degree that may be connected to other subnetworks via a linear chain. The resulting subnetworks were used to identify correlated set topics that demonstrate similar behavior over time.

Then, we performed the following posterior simulation to obtain the distribution of topic cluster assignments. Over R=1000 posterior samples, for posterior sample r, for all days $m\in M$ (e.g., day 4), we sampled a distribution of topics $\theta_{m}^{\left( r \right)}$. Then, we sampled a topic assignment $z_{m,n}^{\left( r \right)}\sim\mathrm{Multinomial}(\theta_{m}^{\left( r \right)})$ and recorded $z_{m,n}^{\left( r \right)}$’s corresponding topic cluster defined by the topic-to-topic correlation network. Finally, we sampled a taxonomic unit $\hat{x}_{m,n}^{\left( r \right)}|z_{m,n}^{(r)}\sim Mulinomial(z_{m,n}^{\left( r \right)},\beta)$.

**Assessing performance.** For each topic cluster from each of the 12 trajectory sets, using the posterior distribution for each model fit, we calculated four statistics: cluster purity, cluster F1 score, cluster root mean square error (RMSE), and taxa RMSE. Cluster F1 score is a weighted average of cluster recall and precision. For each cluster c, we calculated the number of topic assignments $z_{m,n}$ belonging to cluster c that were sampled on days m in which SC_w_ was present (true positives), the number of times topic assignments $z_{m,n}$from cluster c were sampled on days m in which SC_w_ was not present (false positives), and the number of times topic assignments $z_{m,n}$ from cluster c were not sampled on days m in which SC_w_ was present (false negatives). F1 score is then defined as $F1=2\times TP/(2\times TP+FP+FN)$. For a subcommunity of interest w, cluster purity represents the proportion of taxonomic units x sampled from a topic belonging to cluster c that are members of SC_w_: p. Purity was averaged over 25 sample batches to assess uncertainty.

Cluster RMSE was calculated as

$$\mathrm{RMS}E_{c}=\sqrt{\frac{1}{D}\sum\left( p\left( c|d \right)-p\left( SC_{w} | d \right) \right)^{2}}$$

where $p\left( c|d \right)$ is the frequency of cluster c being sampled on day d, averaged across 25 batches; $p\left( SC_{w} | d \right)$ is the frequency of SC_w_ on day d in the raw relative abundance table; and D is the length of the time-series. Taxa RMSE was calculated as

$$\mathrm{RMS}E_{x}=\sqrt{\frac{1}{D}\sum\left( p\left( x_{n}\in\mathrm{SC}_{w}|d,c \right)-p\left( SC_{w} | d \right) \right)^{2}}$$

where $p\left( x_{n}\in\mathrm{SC}_{w}|d,c \right)$ is the frequency of the taxa sampled from cluster c belonging to SC_w_ and cluster c being sampled on day d.

**Hierarchical clustering.** We performed hierarchical clustering of taxa on the 12 trajectory sets. This provided results from an alternative approach that we could use to further evaluate the topic model performance. For each synthetic time-series, we centered and scaled each taxonomic feature using the following equation: $x_{d,n}^{*}={(x}_{d,n}-\bar{x_{\cdot,n}})/sd(x_{\cdot.n})$. Then, we calculated Euclidean distance between the hierarchical taxa clustering and the true taxa clustering for each of the 12 trajectory sets. Scaling each as a feature vector allows us to analyze taxonomic abundance variation over time (shape of the signal), as opposed to differences in taxa counts (which could result in a signal amplitude offset). (Note that the library sizes across samples in this simulation were balanced, per the design of the simulation, hence no library size normalization was necessary.) HC was applied to each distance matrix using Ward's minimum distance criterion. The resulting 12 trees were then cut to produce 30 clusters. The nearly optimal choice of 30 clusters was based on each subcommunity containing 8 of the 250 total taxa for a given time-series. Number of clusters is the only free parameter for HC. We explored using DBSCAN as an additional clustering approach, but because it has two free parameters (number of clusters and signal radius), we felt HC was a more straightforward comparison.

Performance was evaluated in terms of purity (defined above) and cluster RMSE:

$$\mathrm{RMS}E_{\mathrm{hc}}=\sqrt{\frac{1}{D}\sum\left( p\left( hc|d \right)-p\left( SC_{w} | d \right) \right)^{2}}$$

**Comparing topic cluster and HC cluster reconstructions to true long-term oscillatory subcommunity patterns.** We compared subcommunities with long-term, oscillating temporal patterns (ts 7, 8, 10; ts 9, sc 2) to their corresponding reconstructions using the clusters identified via HC and the topic model approach. We generate HC clusters and topic clusters to reconstruct subcommunities (each with their own temporal behavior) from the trajectory sets. HC results in clusters composed of distinct sets of non-overlapping taxa. We take the taxa constituting a particular cluster and subset only those taxa from the original synthetic abundance table. We then sum the abundances over samples (time), to generate a column vector of abundances, and then normalize the vector by the vector sum, resulting in the frequency of the subcommunity for each sample (time) (relative abundances).

For topic clusters, we sample, from the posterior distribution, taxonomic units $\hat{x}_{m,n}$ given day m: $\hat{x}_{m,n}|z_{m,n}$. We record which cluster c contains the assigned topics $z_{m,n}$ for all $\hat{x}_{m,n}$ and the total number of taxa N­­_c_ belonging to a given cluster c. Then, we calculate the frequency $f_{n,c}$ of each taxon x_n_ in each cluster c: $f_{n,c}=\frac{1}{N_{c}}\sum_{n=1}^{N_{c}} x_{n,c}$. We identified high frequency taxa as follows. For a cluster c, we sort (decreasing) the vector of posterior taxa frequencies and apply a moving average filter (n=3) to smooth the trend. We then difference the resulting vector twice. The maximum value is a point of rapid change in the slope of the posterior taxa frequencies against their rank. We use the index of this maximum value to delineate high-frequency taxa in a given topic cluster c. We add 3 to the index to account for second order differences and a moving average (n=3). Using this index, we choose all taxa with posterior frequencies $f_{n,c}$ equal to or above the taxon at this index. Then, as we did for the HC clusters, we acquire its total (summed) abundance over time from the synthetic abundance table and normalize this vector by the vector sum. For the truth, for a given subcommunity, we simply use the subcommunity taxa to subset the synthetic abundance table, calculate its total abundance, and again normalize by the vector sum. Finally, to calculate the mean absolute residual error, we calculate the following:

$$MAE_{m}=\left| y_{t}^{\left( sc \right)}-\hat{y}_{t}^{\left( m \right)} \right|$$

where $\hat{y}$ represents the reconstruction (m) using either HC clusters or topic clusters at time point (sample) t, and sc indicates the true vector of subcommunity abundances.

**REFERENCES**

1. Ditzler G, Rosen G, Polikar R. Information theoretic feature selection for high dimensional metagenomic data. Proceedings - IEEE International Workshop on Genomic Signal Processing and Statistics. 2012. pp. 143–146. doi:10.1109/GENSIPS.2012.6507749

2. Ditzler G, Morrison JC, Lan Y, Rosen GL. Fizzy: feature subset selection for metagenomics. BMC Bioinformatics. BMC Bioinformatics; 2015;16: 358. doi:10.1186/s12859-015-0793-8

3. Karpinets T V, Gopalakrishnan V, Wargo J, Futreal AP, Schadt CW, Zhang J. Linking associations of rare low-abundance species to their environments by association networks. Front Microbiol. 2018;9: 297. doi:10.3389/FMICB.2018.00297

4. Gloor GB, Reid G. Compositional analysis: a valid approach to analyze microbiome high throughput sequencing data. Can J Microbiol. 2016;703: cjm-2015-0821. doi:10.1139/cjm-2015-0821

5. Tjalsma H, Boleij A, Marchesi JR, Dutilh BE. A bacterial driver-passenger model for colorectal cancer: beyond the usual suspects. Nat Rev Microbiol. Nature Publishing Group; 2012;10: 575–82. doi:10.1038/nrmicro2819

6. Lewis JD, Chen EZ, Baldassano RN, Otley AR, Griffiths AM, Lee D, et al. Inflammation, Antibiotics, and Diet as Environmental Stressors of the Gut Microbiome in Pediatric Crohn’s Disease. Cell Host Microbe. Elsevier Inc.; 2015;18: 489–500. doi:10.1016/j.chom.2015.09.008

7. Weiss SJ, Xu Z, Amir A, Peddada S, Bittinger K, Gonzalez A, et al. Effects of library size variance, sparsity, and compositionality on the analysis of microbiome data. PeerJ. 2015;230313: 1–17. doi:https://dx.doi.org/10.7287/peerj.preprints.1157v1

8. Lundin D, Severin I, Logue JB, Östman Ö, Andersson AF, Lindström ES. Which sequencing depth is sufficient to describe patterns in bacterial ??- and ??-diversity? Environ Microbiol Rep. 2012;4: 367–372. doi:10.1111/j.1758-2229.2012.00345.x

9. Smith DP, Peay KG. Sequence depth, not PCR replication, improves ecological inference from next generation DNA sequencing. PLoS One. 2014;9. doi:10.1371/journal.pone.0090234

10. Kuczynski J, Liu Z, Lozupone C, McDonald D, Fierer N, Knight R. Microbial community resemblance methods differ in their ability to detect biologically relevant patterns. Nat Methods. 2010;7: 813–819. doi:10.1038/nmeth.1499

11. Caporaso JG, Lauber CL, Walters WA, Berg-Lyons D, Lozupone CA, Turnbaugh PJ, et al. Global patterns of 16S rRNA diversity at a depth of millions of sequences per sample. Proc Natl Acad Sci. 2011;108: 4516–4522. doi:10.1073/pnas.1000080107

12. Oono R. A confidence interval analysis of sampling effort, sequencing depth, and taxonomic resolution of fungal community ecology in the era of high-throughput sequencing. PLoS One. 2017;12. doi:10.1371/journal.pone.0189796

13. Weiss S, Xu ZZ, Peddada S, Amir A, Bittinger K, Gonzalez A, et al. Normalization and microbial differential abundance strategies depend upon data characteristics. Microbiome. Microbiome; 2017;5: 27. doi:10.1186/s40168-017-0237-y

14. Silverman JD, Washburne AD, Mukherjee S, David LA. A phylogenetic transform enhances analysis of compositional microbiota data. Elife. 2017;6. doi:10.7554/eLife.21887

15. Roberts, Margaret E., Stewart BM, Tingley D. stm: R Package for Structural Topic Models [Internet]. 2017. Available: http://www.structuraltopicmodel.com.

16. Oksanen J, Blanchet FG, Kindt R, Legendre P, Minchin PR, O’Hara RB, et al. vegan: Community Ecology Package [Internet]. R package version 2.3-1. 2015. p. 264. doi:10.4135/9781412971874.n145

17. Gevers D, Kugathasan S, Denson LA, Vázquez-Baeza Y, Van Treuren W, Ren B, et al. The Treatment-Naive Microbiome in New-Onset Crohn’s Disease. Cell Host Microbe. 2014;15: 382–392. doi:10.1016/j.chom.2014.02.005

18. Xu L, Paterson AD, Turpin W, Xu W. Assessment and Selection of Competing Models for Zero-Inflated Microbiome Data. PLoS One. 2015;10: e0129606. doi:10.1371/journal.pone.0129606

19. Love MI, Anders S, Huber W. Differential analysis of count data - the DESeq2 package [Internet]. Genome Biology. 2014. doi:110.1186/s13059-014-0550-8

20. Hall MW, Rohwer RR, Perrie J, McMahon KD, Beiko RG. Ananke: Temporal clustering reveals ecological dynamics of microbial communities. 2017; doi:10.7287/PEERJ.PREPRINTS.2879V1

21. Zhao T, Liu H, Roeder K. The huge package for high-dimensional undirected graph estimation in r. J Mach …. 2012;13: 1059–1062. doi:10.1002/aur.1474.Replication

22. Woloszynek S, Mell JC, Simpson G, O&#039;Connor MP, Rosen GL. Uncovering thematic structure to link co-occurring taxa and predicted functional content in 16S rRNA marker gene surveys. bioRxiv. 2017; Available: http://biorxiv.org/content/early/2017/06/18/146126.abstract

23. Meinshausen N, Bühlmann P. High Dimensional Graphs and Variable Selection with the Lasso. Ann Stat. 2006;34: 1436–1462. doi:10.1214/009053606000000281

**FIGURES**


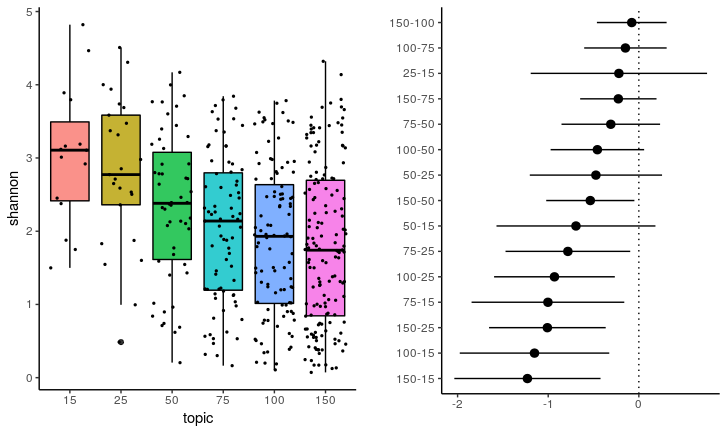


Figure S1. Left: The distribution of Shannon entropy for a variety of topic models of different sizes fit on Gevers data. Each point represents a sample. Right: The adjusted mean differences in Shannon entropy between different topic models via TukeyHSD; 95% adjusted confidence intervals are shown.


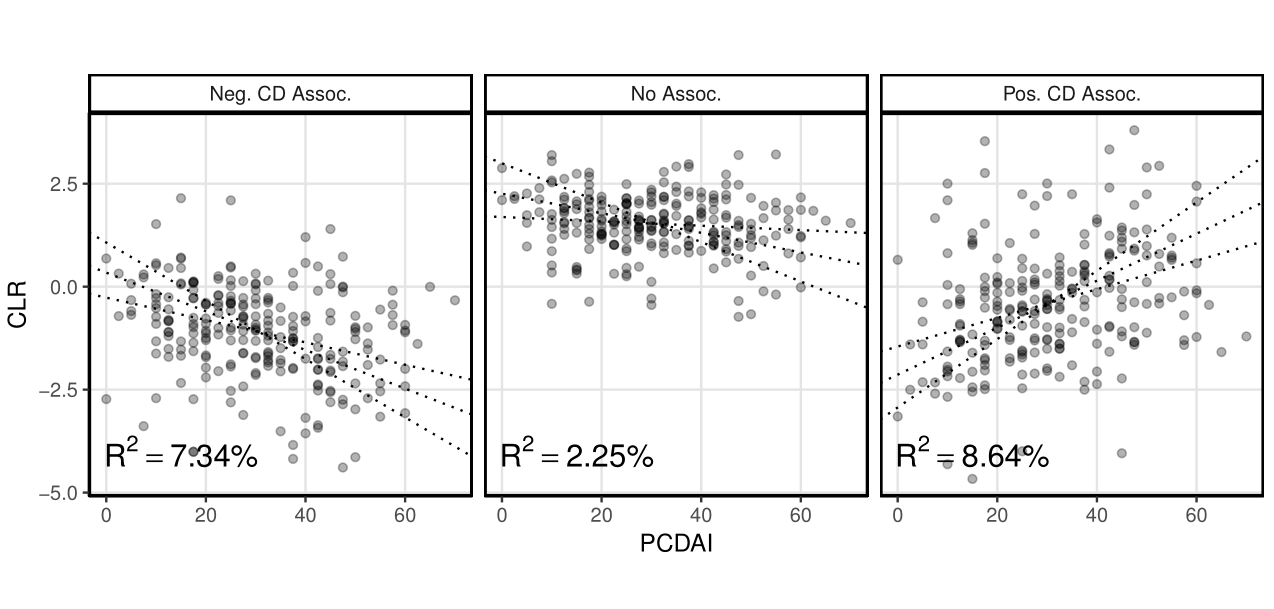


Figure S2. Reduced major axis regression between PCDAI score and the relative abundance of high frequency taxa from high ranking topics (center log ratio transformed).


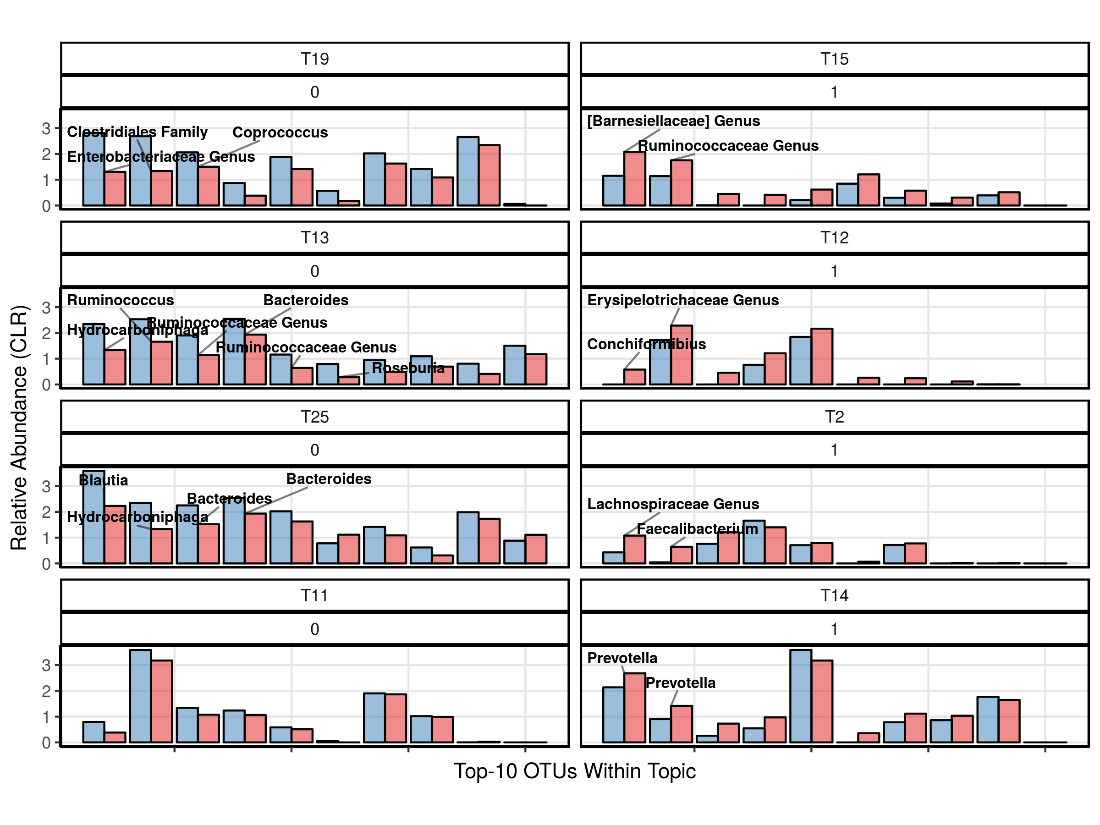


Figure S3. Taxonomic (centered-log-ratio transformed) relative abundances pertaining to top-10 highest frequency taxa from high-ranking topics. Columns correspond CD- (0) associated topics (T19, T13, T25, T11) and CD+ (1) associated topics (T15, T12, T2, T14). Each panel contains 10 pairs of blue and red bars, where each bar represents one of the top-10 taxa for that topic, as well as the prevalence of CLR-transformed taxa in CD- and CD+ samples, respectively.


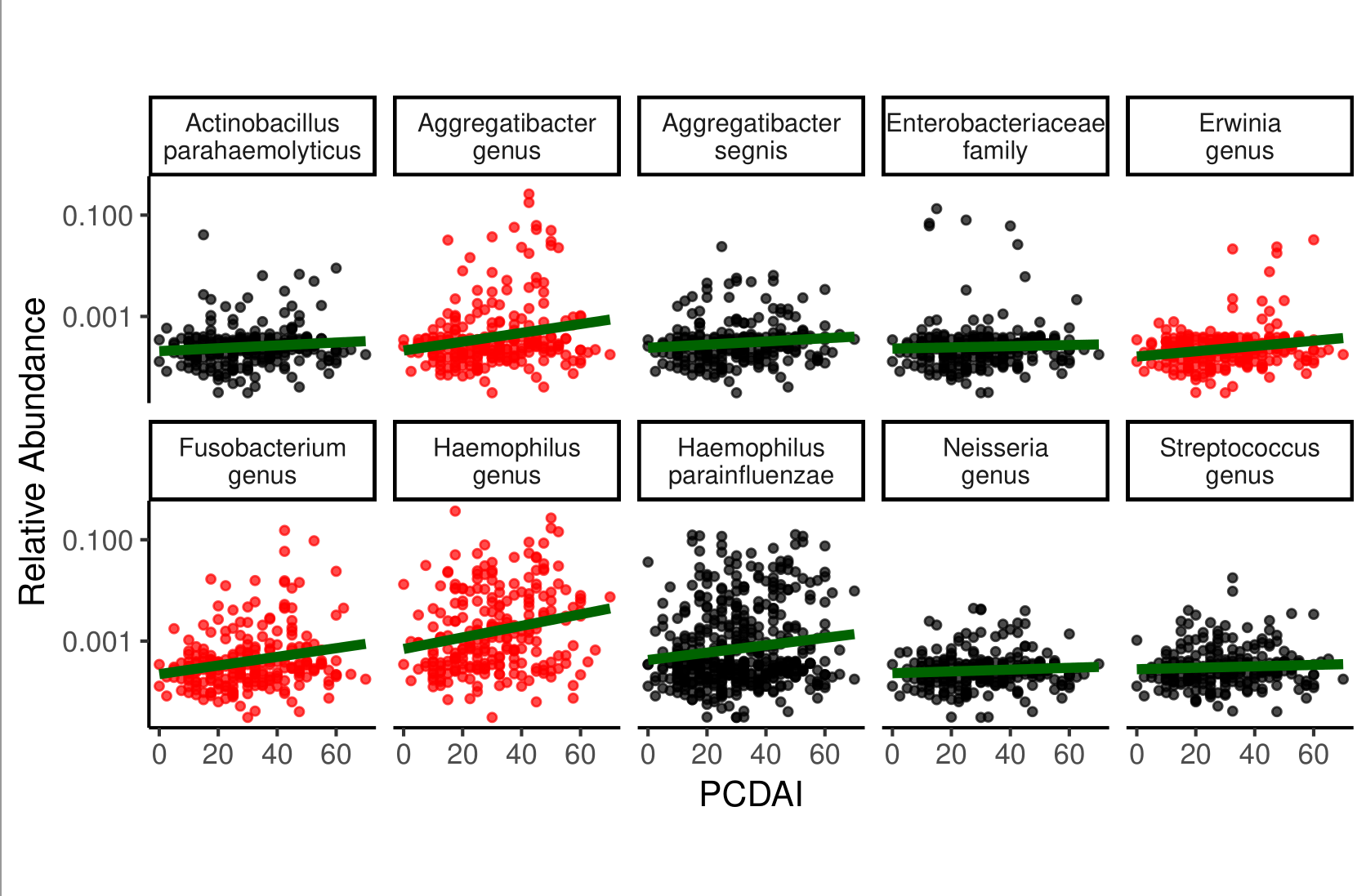


Figure S4. Scatterplots of Gevers data for the relative abundance of taxa that comprise a high frequency cluster in T15 versus PCDAI, a clinical measure of CD disease burden. Red points reflect significance (p < 0.05) via negative binomial regression (log linked, sample library size offset) with Bonferroni correction.


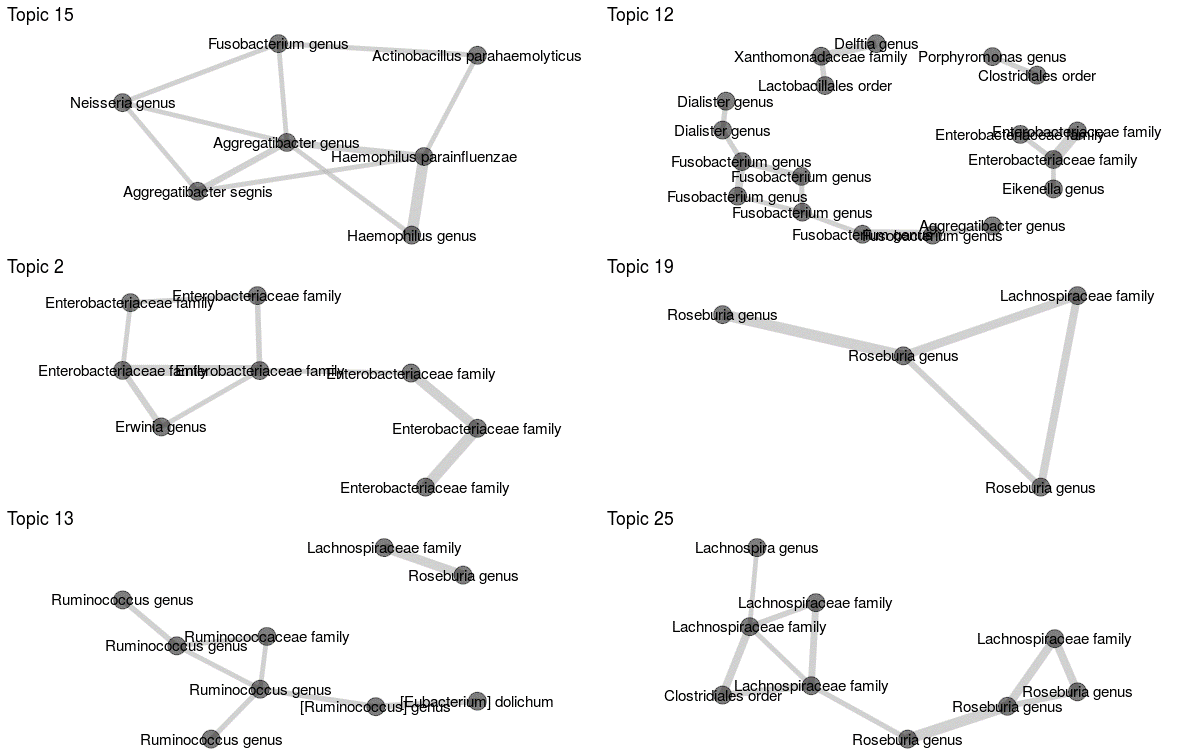


Figure S5. Weighted graph via the sparse neighborhood SPIEC-EASI procedure. Zero weight edges are omitted. Topics correspond to top 3 topics most associated with CD+ and CD-, whereas the taxa within a given topic correspond with “topic clusters of interest,” identified via hierarchically clustering the topics over OTUs distribution (Wards method on Bray Curtis distances).


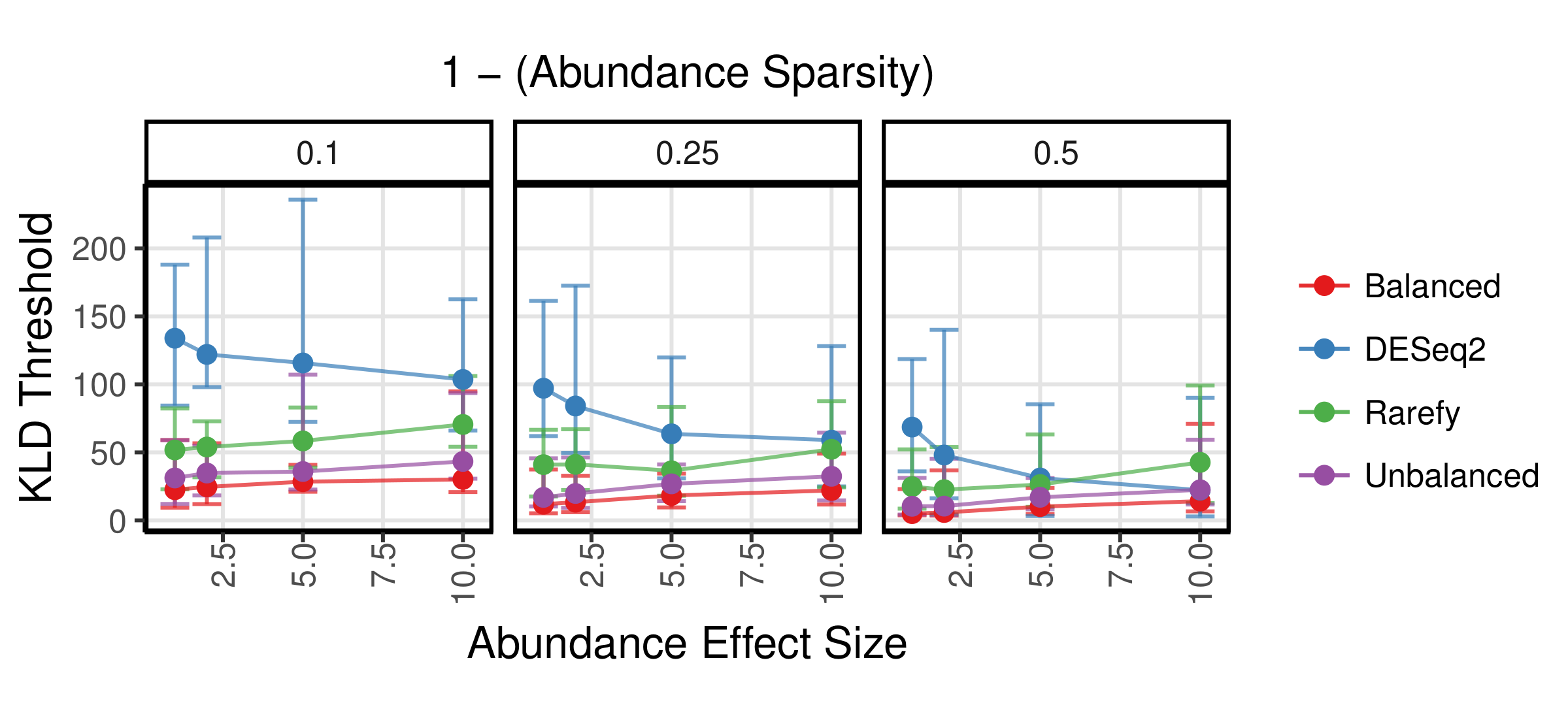


Figure S6. Simulation 1 (K25) KLD threshold scores as a function of subcommunity effect size, where the smaller the KLD means that the topic model approximated the true community structure better. Simulated data consisted of 100 samples across 500 taxonomic features. Panel columns are ordered (left to right) in terms of decreasing sparsity (1-sc_p_). The KLD values are averaged across results from the following simulation parameterizations: proportion of samples containing subcommunities (0.25, 0.50, 0.75) and number of taxa in a subcommunity (10, 15, 30). Vertical bars indicate the range of the KLD threshold score across those parameterizations. Colors represent normalization method, where “balanced” are the simulated absolute abundances and “unbalanced” are the abundances after resampling with respect to library size. Small KLD threshold scores imply high correspondence between p(x_n_|SC_w_)_data_ and p(x_n_|SC_w_,k)_model_. For simulations where the taxa occurrences are sparse, DESeq2 normalization results in poorer topic-SC mappings (high threshold scores), but as sparsity decreases the differences between normalization methods become less pronounced.


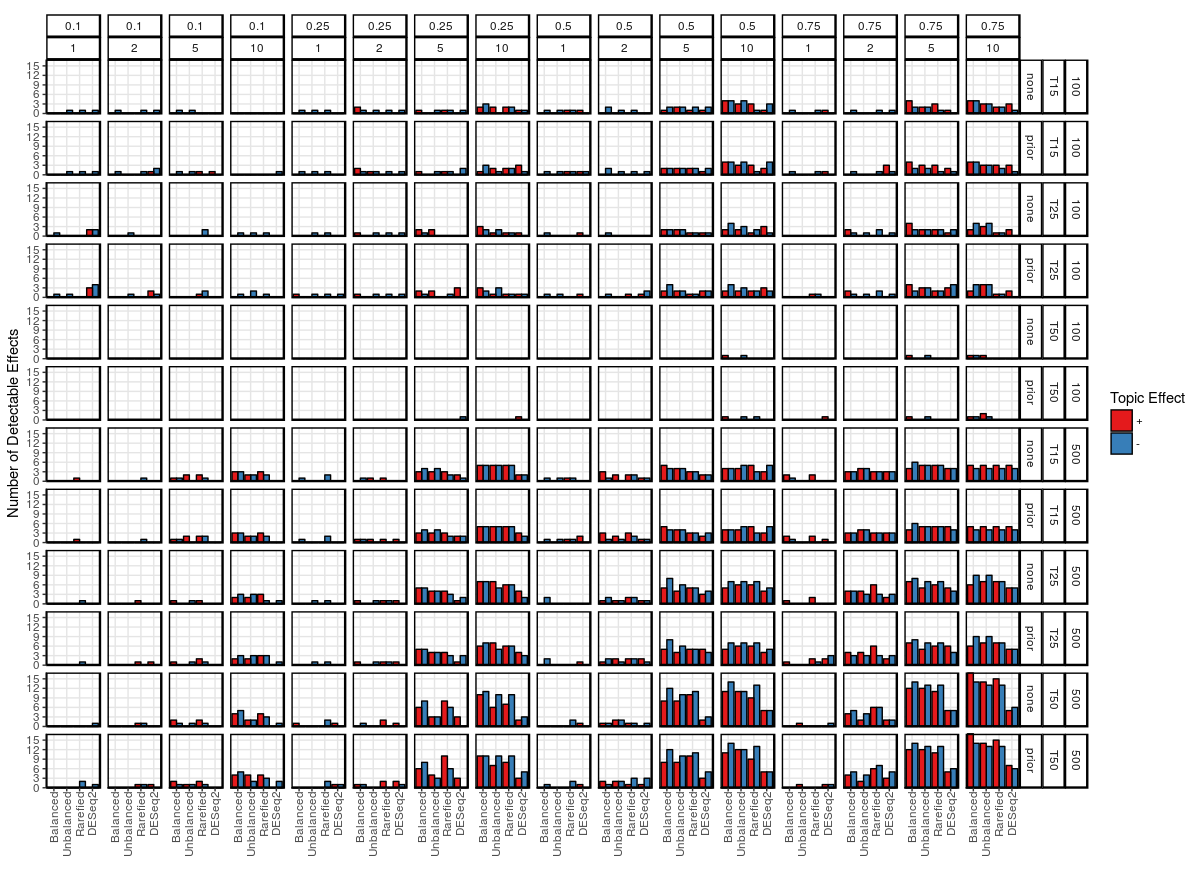


Figure S7. Simulation 1 detected effects as a function of normalization method. Panel rows are ordered in terms (1) presence of prior information (binary indicator for treatment group), (2) number of topics, and (3) sample size (100 samples, 500 taxonomic features; 500 samples, 1000 taxonomic features). Panel columns are arranged in terms of decreasing SC sparsity (1-sc_p_) (top) and SC effect size (bottom). Bars are colored based on the direction of the detectable effects, where positive effects (associated with the treatment group) and negative effects are red and blue, respectively. We consider results for balanced data (absolute abundances) as a best-case-scenario; hence, significant deviations from the effects detected for balanced data would suggest poor performance in terms of type 1 or type 2 errors.


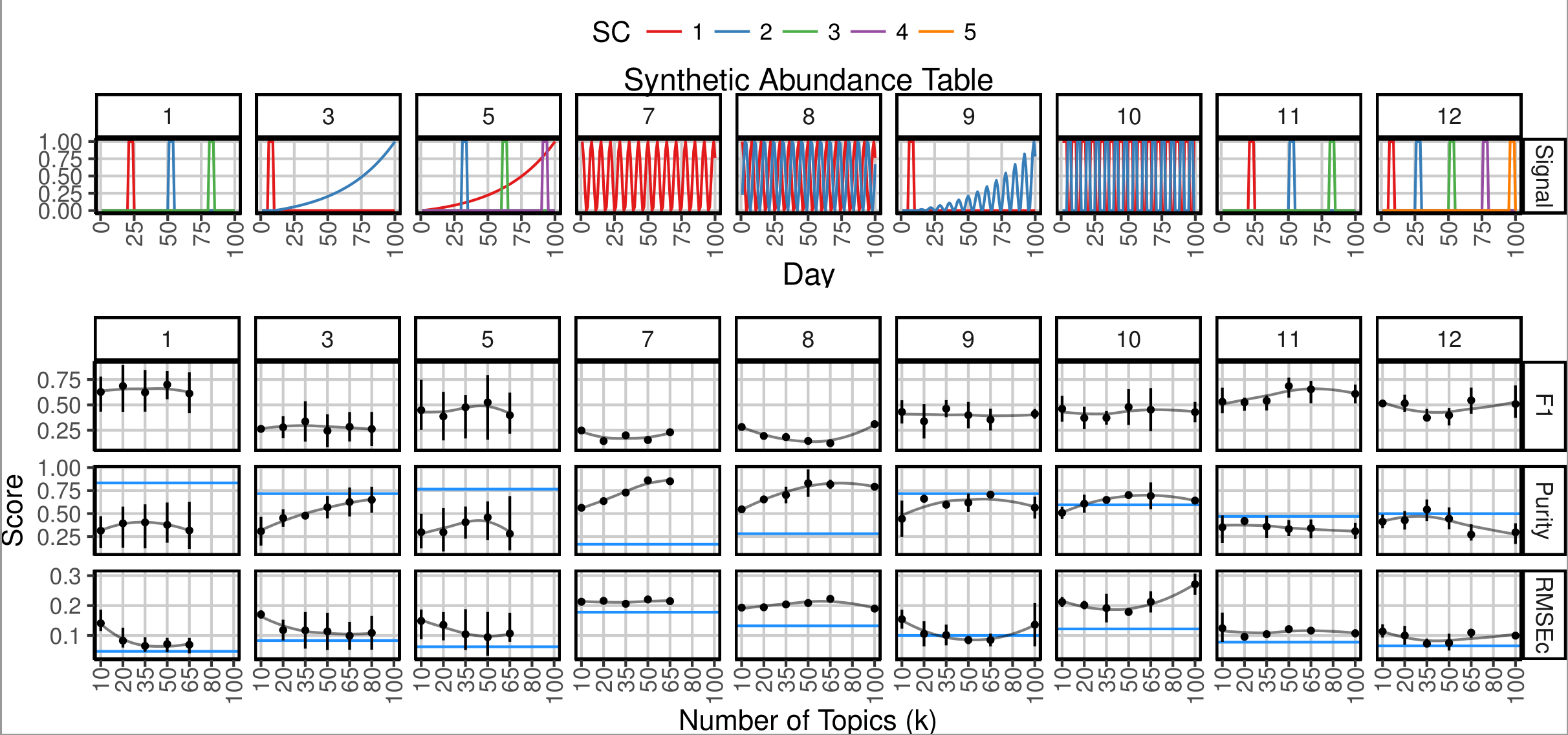


Figure S8. (Top) Simulation 2 patterns applied to nine of the twelve synthetic taxonomic abundance tables (100 samples, 250 taxa). Each pattern was used to generate a specific subcommunity (a subset of taxa that co-occurred over time). A set of patterns sharing a background distribution (each facet) is termed a trajectory set. (Bottom) The performance scores as a function of the number of topics in a model. Rows contain the performance scores F1, purity, and cluster RMSE. Columns contain the scores pertaining to a specific trajectory set. Colors correspond to a given SC. Scores (purity and cluster RMSE) for the performance of hierarchical clustering (k=30) are marked by horizontal blue lines. Both methods had difficulty representing periodic signals (trajectory sets 7-10). Cluster purity was larger for HC clusters, but the difference became negligible for overlapping and periodic time-series.


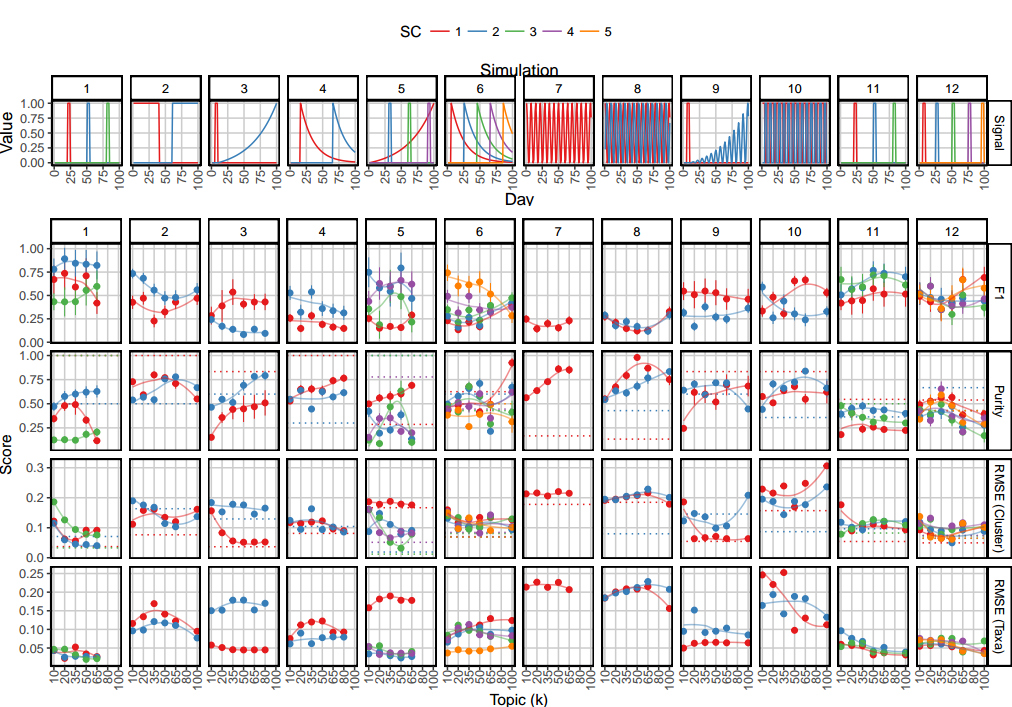


Figure S9. Simulation 2 interventions used to agitate the twelve $100\times250$ background distributions (top) and performance scores as a function of STM topic number (bottom). Panel rows contain the performance scores F1, purity, cluster RMSE and taxa RMSE. Panel columns contain the scores for each synthetic time-series after the corresponding interventions were applied. Colors correspond to a given SC. Hierarchical clustering (k=30) RMSE and purity scores from top performing clusters are shown as horizontal dotted lines. Performance suffered for periodic signals (time-series 7-10). Cluster purity was larger for HC clusters, but the difference became negligible for overlapping and periodic time-series.


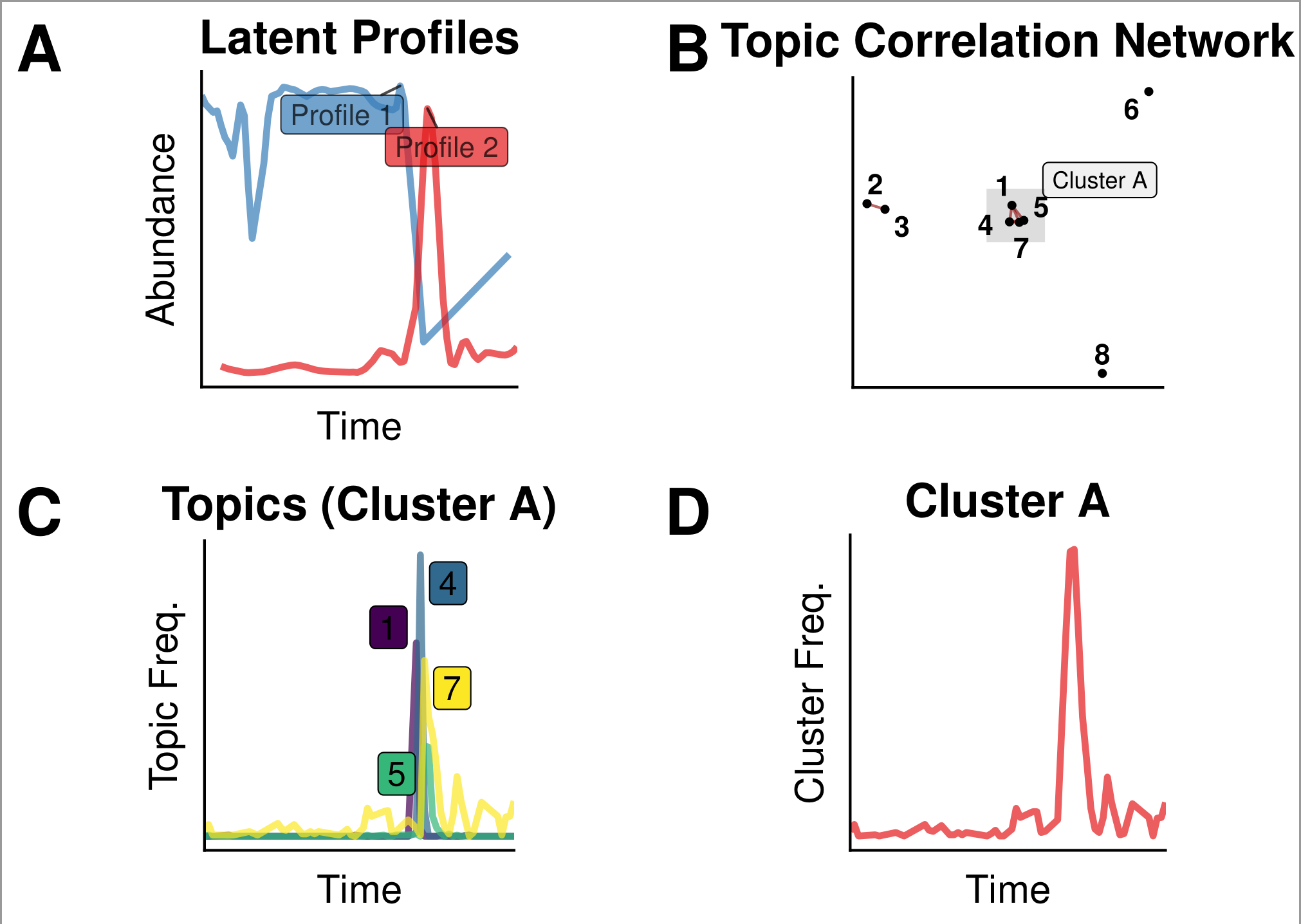


Figure S10. Diagram showing our approach to uncover temporal signals using topics. (A) Given two latent taxonomic abundance profiles that we wish to capture, each which represent co-occurring sets of taxa that behave similarly over time, we build a correlation network of topics (B) to identify topics, composed of multiple taxa, that co-occur across samples (2, 3 and 1, 4, 5, 7). In (C), focusing on cluster A (topics 1, 5, 7, 9), we can see that their abundances are similar to profile 2. When summed together, they reconstruct profile 2 (D).


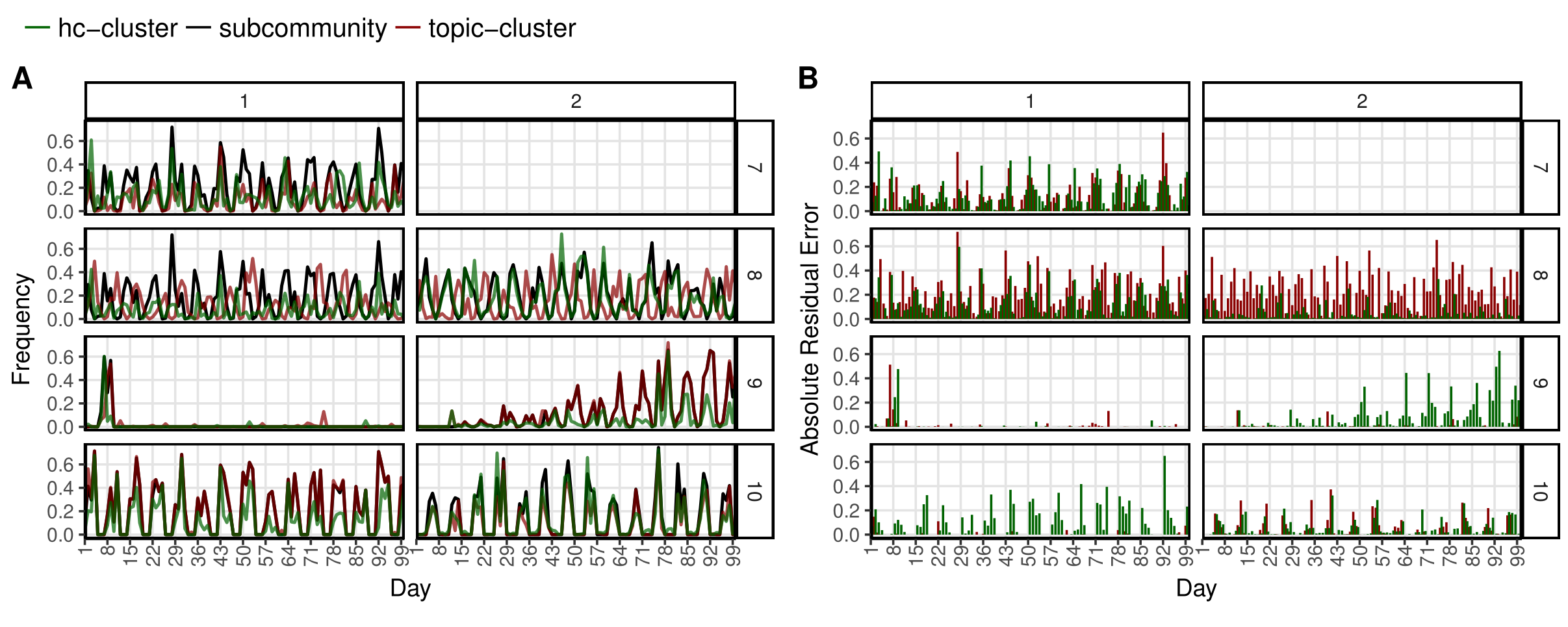
Figure S11. (A) The time series trajectories (relative abundances) over time for oscillatory trajectory sets 7-10 (row). Each subcommunity in a given trajectory set are shown in its own column (ts 7 had only one subcommunity). The true subcommunity trajectory is shown in black. The trajectory of the taxa identified by the topic model approach and HC to represent its corresponding subcommunity are shown in red and green, respectively. (B) The mean absolute error between the true subcommunity trajectory and the HC reconstruction (green) and the topic model approach reconstruction (red). The figure suggest that HC had more difficulty identifying the taxa comprising the subcommunities in trajectory set 9 and subcommunity 1 in trajectory set 10, whereas the topic model has more difficult with subcommunity 2 in trajectory set 8.


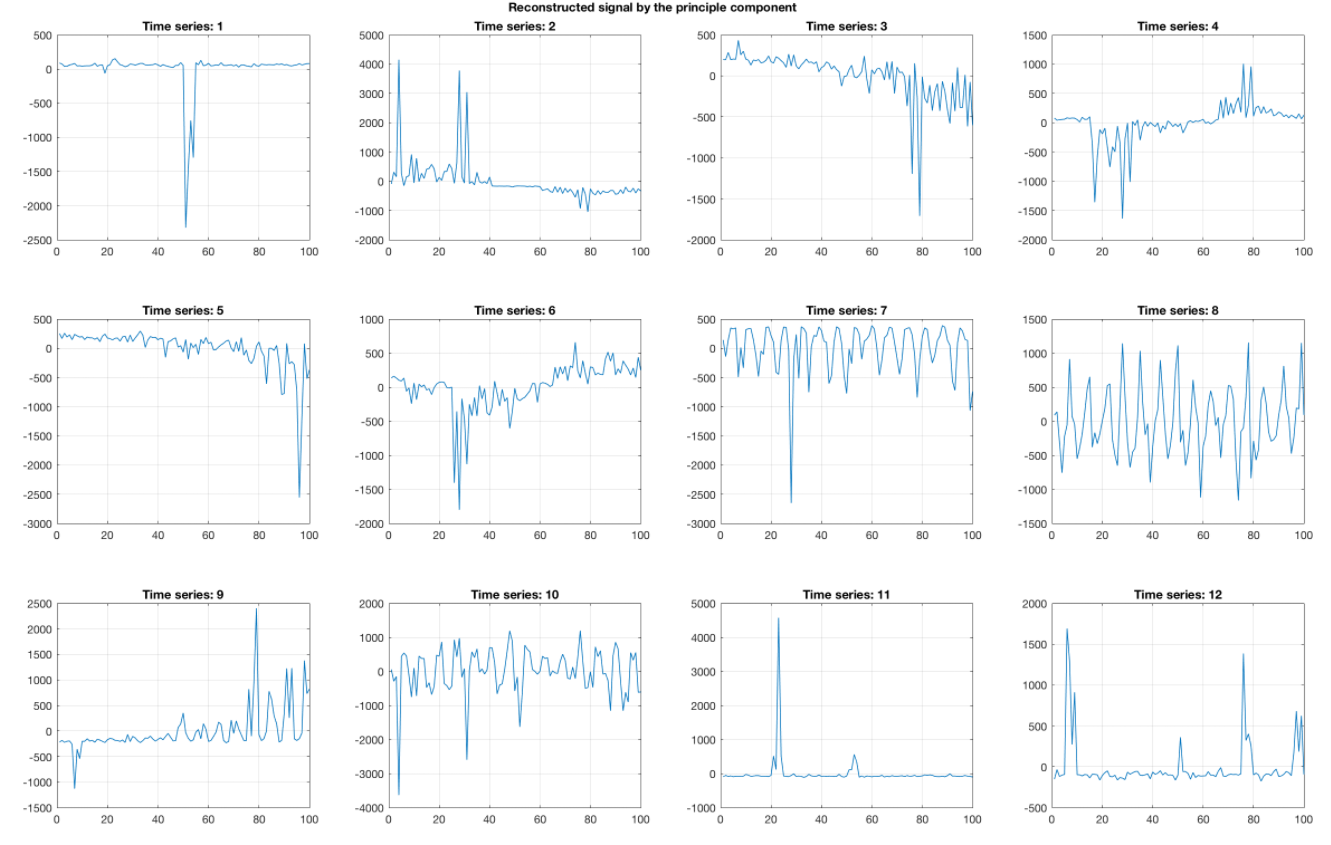


S12 Fig. PCA reconstruction of simulation 2.


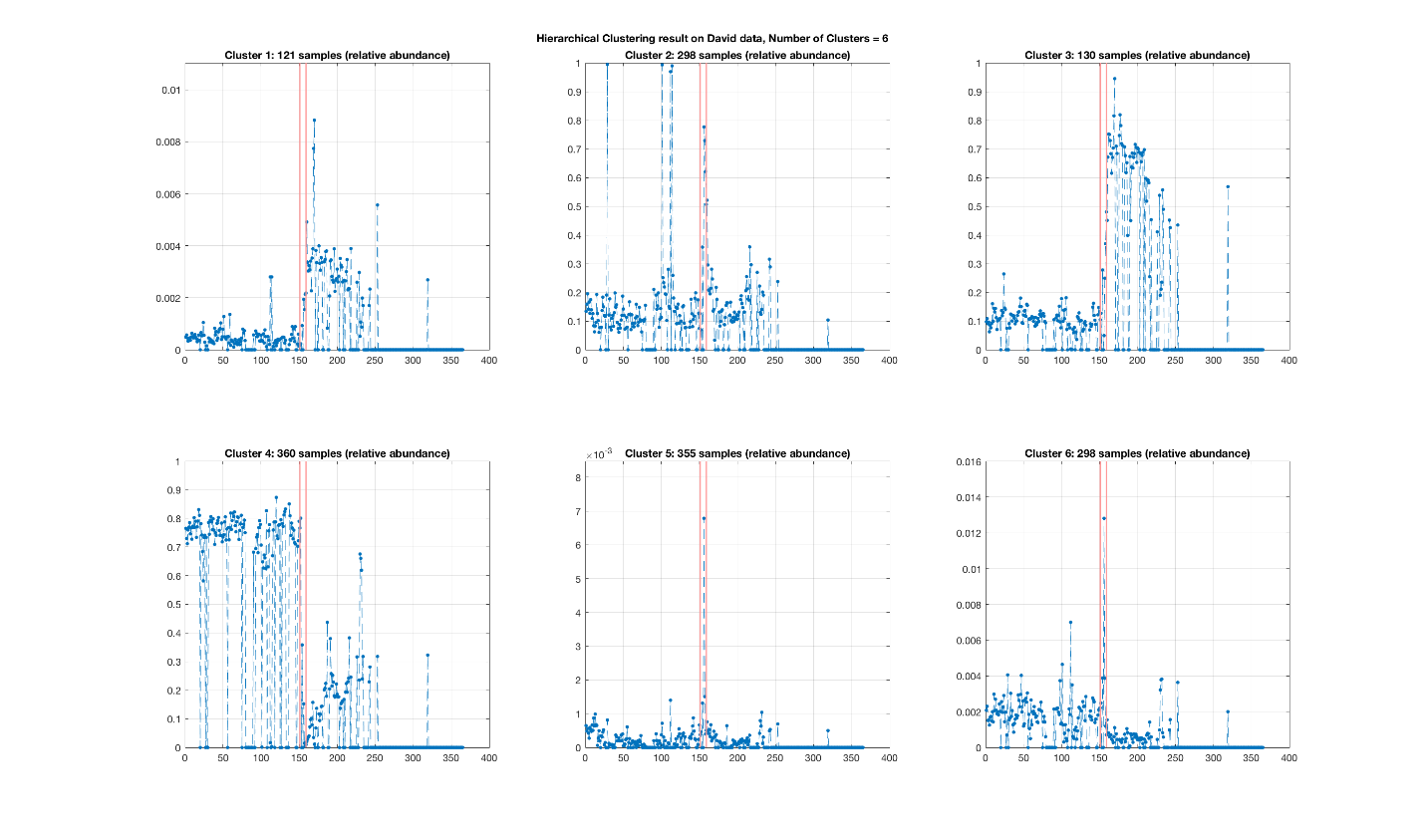


S13 Fig. Clusters via hierarchical clustering (k=6) applied to the David et al. dataset (subset B). Red lines signify the presentation of illness.


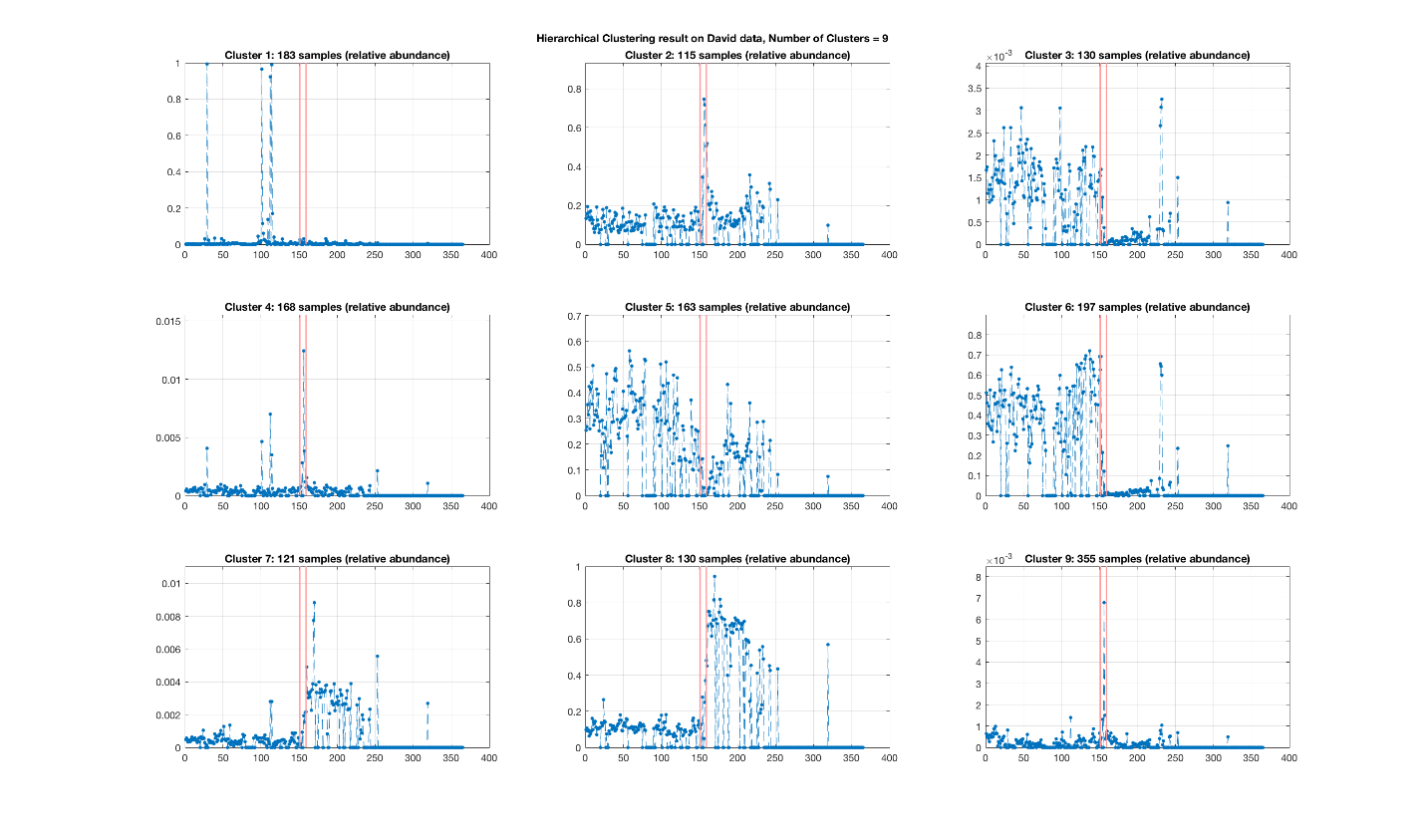


S14 Fig. Clusters via hierarchical clustering (k=9) applied to the David et al. dataset (subset B). Red lines signify the presentation of illness.


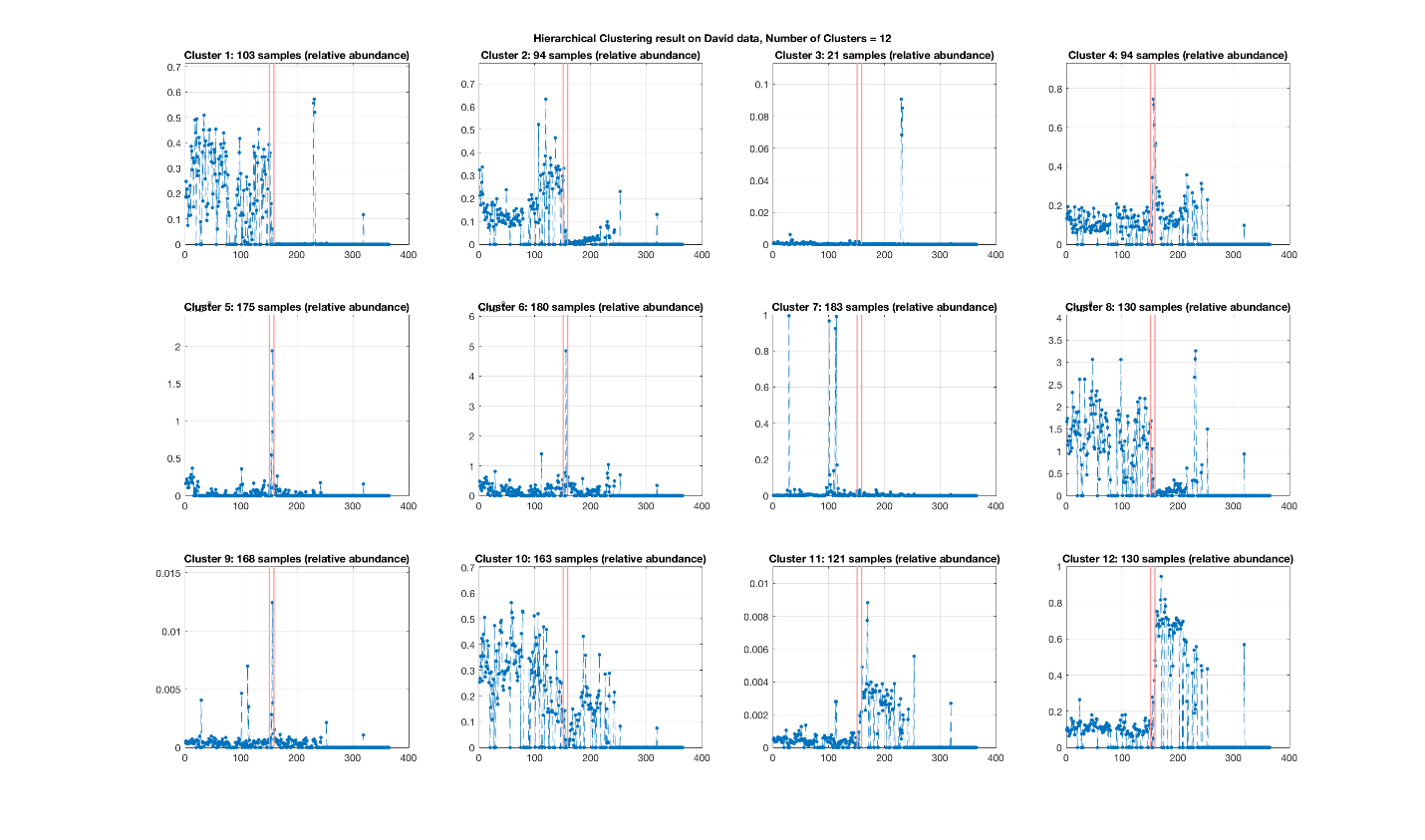


S15 Fig. Clusters via hierarchical clustering (k=12) applied to the David et al. dataset (subset B). Red lines signify the presentation of illness.


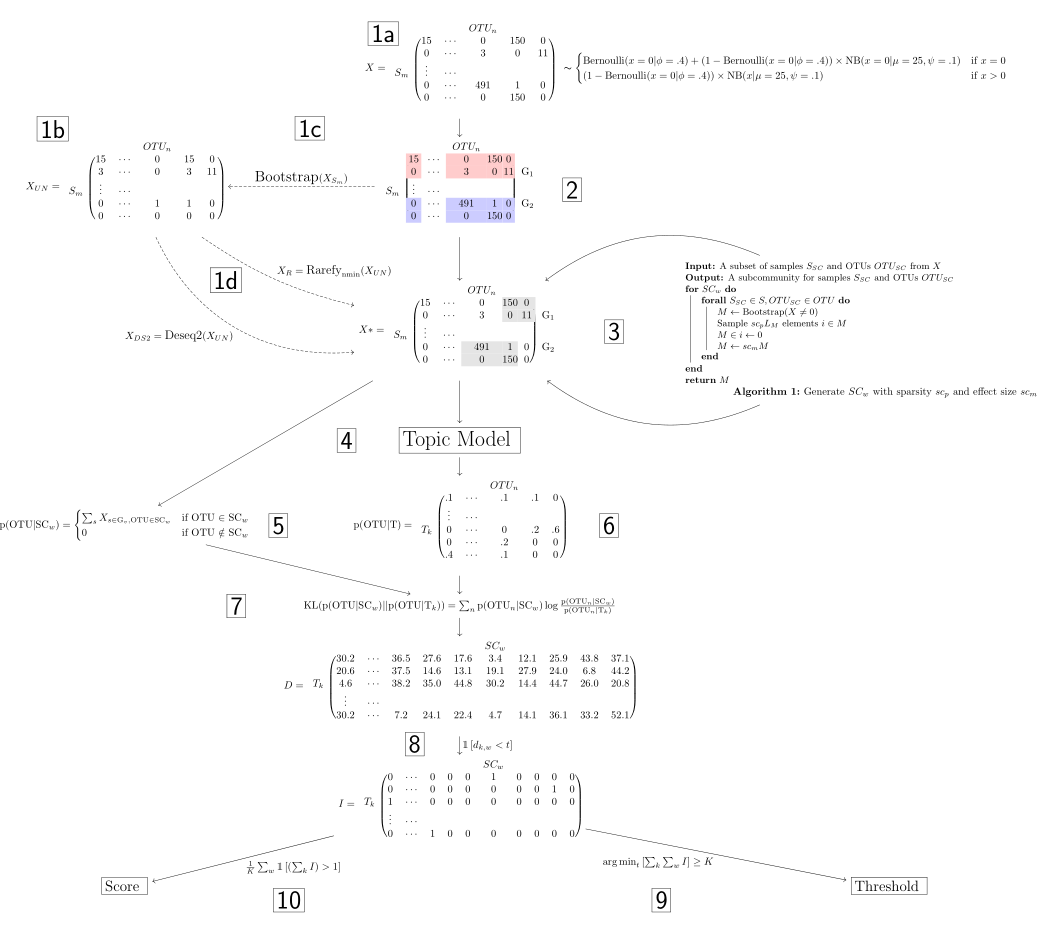


S16 Fig. Workflow for simulation 1. (1a) A background distribution generated from a zero-inflated negative binomial distribution (ZINB) with sparsity (φ), mean (μ), and size (ψ) parameters. (2) Samples were randomly split into treatment (G1) and control (G2) groups. (3) 15 subcommunities (SCs) of size sc_l_ ∈ (10, 15, 30) were generated by resampling with replacement all nonzero values in the background distribution and then scaling these values by effect size sc_m_ ∈ (1, 2, 5, 10) and setting a proportion 1-sc_p_ (sc_p_ ∈ (0.10, 0.25, 0.5,.0.75)) of these values to zero. 5 SCs each were set to replace the taxa abundances from a proportion g_p_ ∈ (0.25, 0.50, 0.75) treatment samples, control samples, and an equal proportion from both treatment and control samples. (1b) Library sizes for each sample were randomly generated from a discrete uniform distribution [100, min(sample sum)] and used to resample the background distribution. (1c,d) This table was then either rarefied to a balanced library size (1000) or normalized using the DESeq2 variance stabilizing transformation to create the two additional abundance tables. (4) STMs were fit. (5-7) We calculated Kullback-Leibler divergence (KLD) between p(x_n_|SC_w_)_data_ and p(x_n_|SC_w_,k)_model_, resulting in a distance for each topic-SC pair for a given model parameterization. (8-9) For a given STM with K topics, we identified the minimum threshold th in which there remain K KLD values less than th. (10) We summed the number of SCs to which each of these K topics mapped (“redundancy scores”).


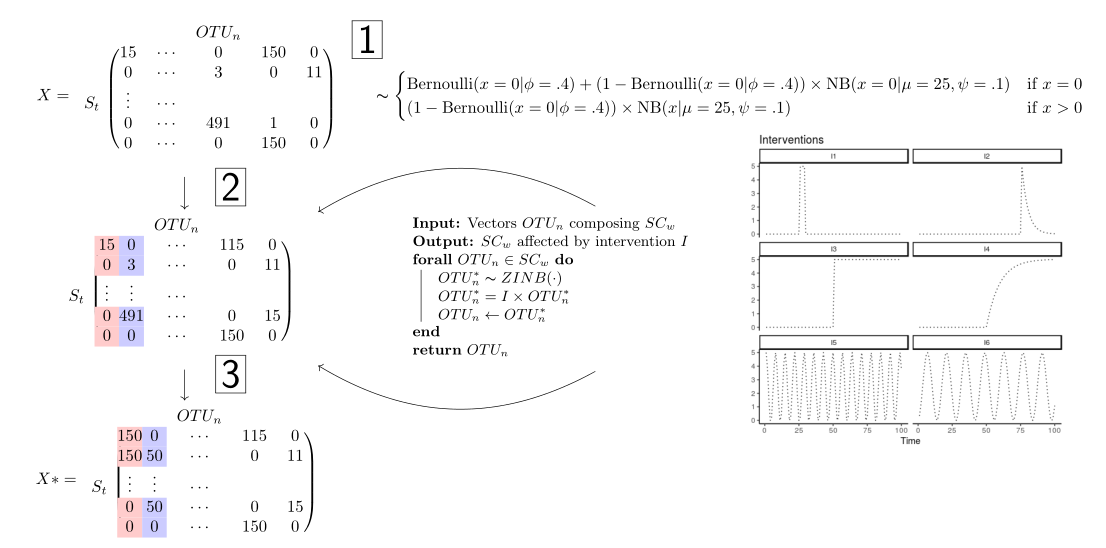


S17 Fig. Workflow for simulation 2. (1) 12 background distributions were generated from a zero-inflated negative binomial distribution (ZINB) with sparsity (φ), mean (μ), and size (ψ) parameters. (2-3) Each SC of 8 taxa were agitated with one or more interventions: pulses (I1, I2), steps (I3, I4), or periodicity (I5, I6).

**TABLES**


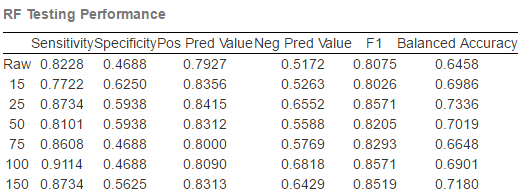


Table S1. Generalization error for Gevers data during testing from repeated (10x) 10-fold CV with up-sampling, using an RF classifier. Performance of OTU relative abundances in designated as “Raw,” whereas numbers represent the number of topics in a given model. No sample information was used for topic model fitting. Training-testing set splits were consistent across models.


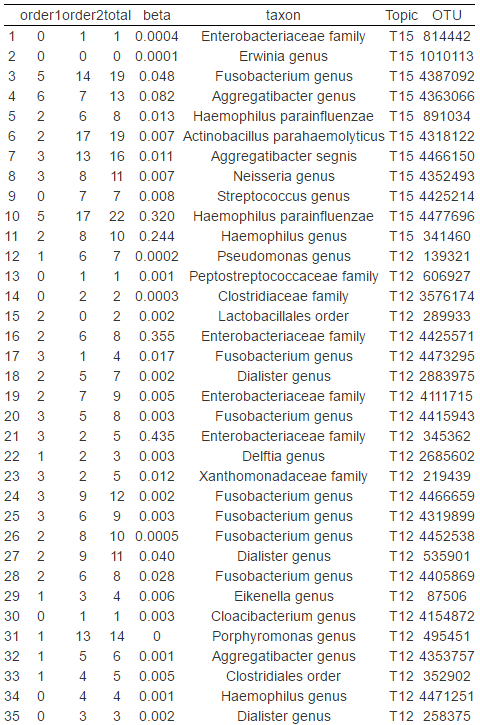


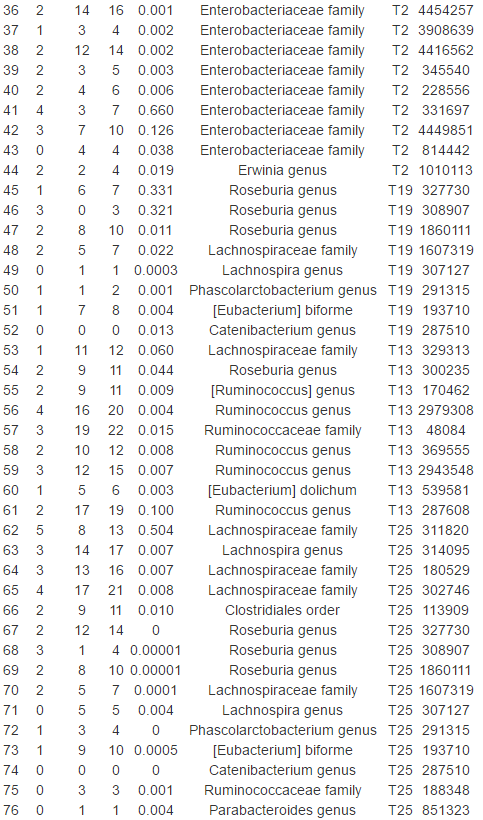


Table S2. Values for the topics over OTUs distribution (beta) for “topic clusters of interest,” identified via hierarchical clustering (Wards method on Bray Curtis distances), alongside the number of first order connections (direct connections to other taxa within the cluster, OTU_c_-OTU_c’_) and second order connections (indirect connections to other taxa within the cluster via an intermediate OTU not present in the cluster, OTU_c_-OTU_nc_-OTU_c’_) from the sparse neighborhood SPIEC-EASI procedure.


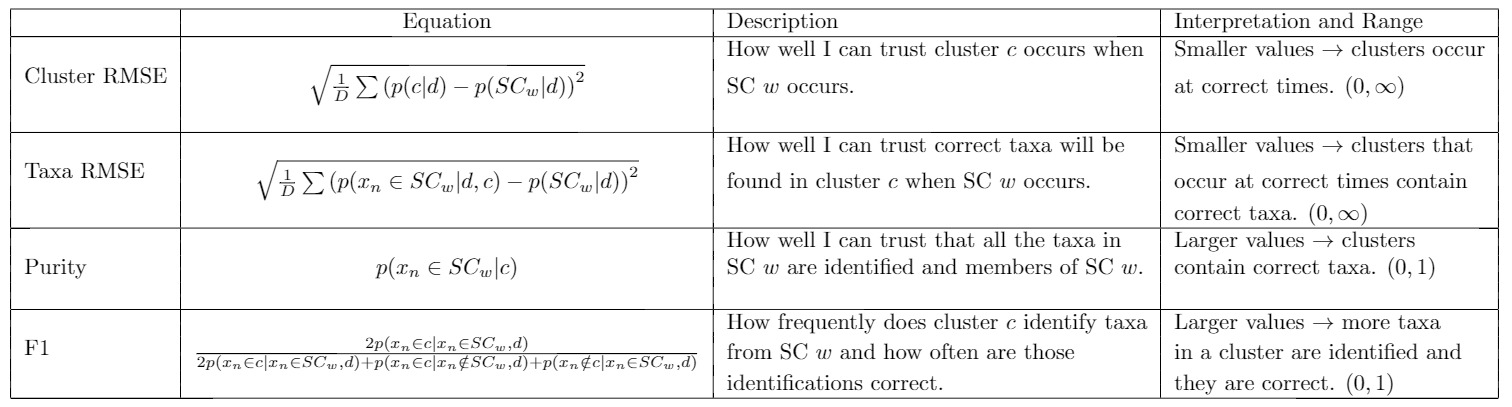


Table S3. Measures of quality for time-series pattern reconstruction

| TS | SC | Statistic | 10 | 20 | 35 | 50 | 65 | 80 | 100 |
| --- | --- | --- | --- | --- | --- | --- | --- | --- | --- |
| 1 | 1 | F1 Purity RMSEc RMSEt | 0.67 (0.15) 0.35 (0.04) 0.12 (0.01) 0.04 (0.01) | 0.74 (0.18) 0.48 (0.05) 0.06 (0.01) 0.02 (0.01) | 0.59 (0.16) 0.49 (0.10) 0.06 (0.01) 0.05 (0.00) | 0.71 (0.19) 0.33 (0.04) 0.09 (0.01) 0.03 (0.01) | 0.42 (0.12) 0.12 (0.02) 0.09 (0.00) 0.03 (0.01) | - | - |
| 1 | 2 | F1 Purity RMSEc RMSEt | 0.78 (0.12) 0.47 (0.05) 0.11 (0.01) 0.04 (0.01) | 0.89 (0.12) 0.58 (0.06) 0.06 (0.01) 0.03 (0.01) | 0.84 (0.14) 0.60 (0.05) 0.05 (0.01) 0.03 (0.01) | 0.83 (0.15) 0.62 (0.06) 0.04 (0.01) 0.03 (0.01) | 0.82 (0.15) 0.63 (0.06) 0.04 (0.01) 0.03 (0.01) | - | - |
| 1 | 3 | F1 Purity RMSEc RMSEt | 0.43 (0.09) 0.13 (0.03) 0.19 (0.01) 0.05 (0.01) | 0.43 (0.16) 0.13 (0.04) 0.13 (0.01) 0.05 (0.00) | 0.43 (0.14) 0.12 (0.03) 0.09 (0.01) 0.03 (0.01) | 0.56 (0.16) 0.18 (0.04) 0.08 (0.00) 0.02 (0.01) | 0.60 (0.17) 0.21 (0.04) 0.08 (0.01) 0.02 (0.01) | - | - |
| 2 | 1 | F1 Purity RMSEc RMSEt | 0.43 (0.07) 0.73 (0.02) 0.11 (0.00) 0.12 (0.01) | 0.47 (0.07) 0.59 (0.03) 0.16 (0.01) 0.13 (0.01) | 0.23 (0.07) 0.80 (0.04) 0.16 (0.00) 0.17 (0.00) | 0.32 (0.07) 0.77 (0.03) 0.13 (0.00) 0.14 (0.00) | 0.43 (0.07) 0.71 (0.05) 0.12 (0.01) 0.12 (0.01) | - | 0.47 (0.07) 0.55 (0.02) 0.16 (0.01) 0.09 (0.01) |
| 2 | 2 | F1 Purity RMSEc RMSEt | 0.73 (0.05) 0.54 (0.02) 0.19 (0.01) 0.10 (0.01) | 0.68 (0.06) 0.57 (0.02) 0.18 (0.01) 0.10 (0.01) | 0.56 (0.07) 0.54 (0.02) 0.17 (0.01) 0.12 (0.01) | 0.47 (0.07) 0.76 (0.02) 0.11 (0.01) 0.12 (0.01) | 0.48 (0.08) 0.78 (0.02) 0.10 (0.01) 0.11 (0.01) | - | 0.56 (0.07) 0.67 (0.02) 0.14 (0.01) 0.08 (0.01) |
| 3 | 1 | F1 Purity RMSEc RMSEt | 0.28 (0.10) 0.15 (0.02) 0.16 (0.01) 0.06 (0.01) | 0.39 (0.17) 0.36 (0.06) 0.08 (0.01) 0.05 (0.00) | 0.54 (0.14) 0.44 (0.10) 0.06 (0.01) 0.05 (0.00) | 0.41 (0.05) 0.45 (0.14) 0.05 (0.01) 0.04 (0.00) | 0.43 (0.08) 0.47 (0.12) 0.05 (0.00) 0.05 (0.00) | 0.43 (0.09) 0.51 (0.13) 0.05 (0.01) 0.04 (0.00) | - |
| 3 | 2 | F1 Purity RMSEc RMSEt | 0.24 (0.04) 0.46 (0.03) 0.18 (0.01) 0.15 (0.00) | 0.17 (0.04) 0.55 (0.03) 0.15 (0.00) 0.15 (0.00) | 0.14 (0.04) 0.51 (0.05) 0.18 (0.00) 0.18 (0.00) | 0.08 (0.03) 0.69 (0.06) 0.18 (0.00) 0.18 (0.00) | 0.14 (0.04) 0.78 (0.03) 0.15 (0.00) 0.15 (0.00) | 0.09 (0.03) 0.79 (0.05) 0.17 (0.00) 0.17 (0.00) | - |
| 4 | 1 | F1 Purity RMSEc RMSEt | 0.26 (0.04) 0.53 (0.03) 0.12 (0.01) 0.08 (0.01) | 0.15 (0.04) 0.65 (0.04) 0.11 (0.00) 0.11 (0.00) | 0.28 (0.04) 0.65 (0.04) 0.12 (0.00) 0.12 (0.00) | 0.19 (0.04) 0.63 (0.05) 0.12 (0.00) 0.12 (0.00) | 0.16 (0.04) 0.74 (0.04) 0.10 (0.01) 0.09 (0.00) | 0.15 (0.04) 0.77 (0.04) 0.09 (0.01) 0.09 (0.00) | - |
| 4 | 2 | F1 Purity RMSEc RMSEt | 0.53 (0.08) 0.55 (0.03) 0.12 (0.01) 0.06 (0.01) | 0.32 (0.08) 0.64 (0.05) 0.09 (0.01) 0.09 (0.00) | 0.54 (0.07) 0.45 (0.03) 0.16 (0.01) 0.06 (0.01) | 0.36 (0.08) 0.63 (0.04) 0.09 (0.01) 0.08 (0.00) | 0.34 (0.08) 0.57 (0.03) 0.10 (0.01) 0.08 (0.01) | 0.31 (0.08) 0.61 (0.04) 0.09 (0.01) 0.08 (0.00) | - |
| 5 | 1 | F1 Purity RMSEc RMSEt | 0.25 (0.04) 0.50 (0.03) 0.19 (0.01) 0.16 (0.00) | 0.15 (0.03) 0.56 (0.04) 0.18 (0.00) 0.18 (0.00) | 0.17 (0.04) 0.58 (0.05) 0.19 (0.00) 0.19 (0.00) | 0.16 (0.03) 0.60 (0.04) 0.18 (0.00) 0.18 (0.00) | 0.29 (0.04) 0.69 (0.04) 0.18 (0.00) 0.18 (0.00) | - | - |
| 5 | 2 | F1 Purity RMSEc RMSEt | 0.75 (0.16) 0.42 (0.06) 0.09 (0.01) 0.03 (0.01) | 0.58 (0.12) 0.19 (0.03) 0.15 (0.01) 0.04 (0.01) | 0.54 (0.16) 0.23 (0.04) 0.11 (0.01) 0.03 (0.01) | 0.79 (0.16) 0.39 (0.05) 0.08 (0.01) 0.02 (0.01) | 0.47 (0.13) 0.13 (0.02) 0.09 (0.01) 0.03 (0.01) | - | - |
| 5 | 3 | F1 Purity RMSEc RMSEt | 0.36 (0.13) 0.12 (0.02) 0.16 (0.01) 0.05 (0.00) | 0.19 (0.15) 0.09 (0.03) 0.13 (0.01) 0.06 (0.00) | 0.60 (0.18) 0.47 (0.07) 0.05 (0.01) 0.03 (0.01) | 0.49 (0.13) 0.63 (0.11) 0.03 (0.01) 0.03 (0.01) | 0.22 (0.11) 0.10 (0.04) 0.08 (0.01) 0.04 (0.00) | - | - |
| 5 | 4 | F1 Purity RMSEc RMSEt | 0.44 (0.11) 0.15 (0.02) 0.16 (0.01) 0.05 (0.01) | 0.63 (0.17) 0.35 (0.05) 0.08 (0.01) 0.03 (0.01) | 0.60 (0.16) 0.35 (0.06) 0.07 (0.01) 0.04 (0.01) | 0.66 (0.14) 0.21 (0.03) 0.09 (0.01) 0.04 (0.01) | 0.62 (0.14) 0.20 (0.03) 0.08 (0.01) 0.04 (0.01) | - | - |
| 6 | 1 | F1 Purity RMSEc RMSEt | 0.23 (0.04) 0.42 (0.03) 0.16 (0.01) 0.08 (0.01) | 0.13 (0.04) 0.48 (0.04) 0.12 (0.00) 0.10 (0.00) | 0.22 (0.04) 0.68 (0.04) 0.10 (0.00) 0.10 (0.00) | 0.17 (0.04) 0.41 (0.04) 0.13 (0.01) 0.11 (0.00) | 0.32 (0.05) 0.49 (0.07) 0.13 (0.00) 0.13 (0.00) | - | 0.40 (0.05) 0.92 (0.05) 0.13 (0.00) 0.12 (0.00) |
| 6 | 2 | F1 Purity RMSEc RMSEt | 0.28 (0.05) 0.50 (0.03) 0.13 (0.01) 0.07 (0.01) | 0.16 (0.04) 0.58 (0.04) 0.10 (0.01) 0.09 (0.00) | 0.27 (0.04) 0.66 (0.06) 0.10 (0.00) 0.10 (0.00) | 0.18 (0.04) 0.71 (0.05) 0.10 (0.00) 0.11 (0.00) | 0.39 (0.05) 0.21 (0.02) 0.14 (0.00) 0.09 (0.00) | - | 0.42 (0.05) 0.67 (0.05) 0.09 (0.00) 0.10 (0.00) |
| 6 | 3 | F1 Purity RMSEc RMSEt | 0.35 (0.05) 0.48 (0.03) 0.14 (0.01) 0.08 (0.01) | 0.21 (0.05) 0.41 (0.03) 0.13 (0.00) 0.11 (0.00) | 0.34 (0.05) 0.68 (0.05) 0.10 (0.01) 0.10 (0.00) | 0.24 (0.05) 0.58 (0.04) 0.10 (0.01) 0.09 (0.00) | 0.39 (0.06) 0.29 (0.07) 0.13 (0.00) 0.08 (0.00) | - | 0.47 (0.06) 0.41 (0.12) 0.13 (0.00) 0.07 (0.00) |
| 6 | 4 | F1 Purity RMSEc RMSEt | 0.49 (0.07) 0.45 (0.02) 0.15 (0.01) 0.07 (0.01) | 0.31 (0.07) 0.52 (0.04) 0.11 (0.01) 0.10 (0.00) | 0.49 (0.07) 0.47 (0.06) 0.13 (0.00) 0.11 (0.00) | 0.34 (0.07) 0.60 (0.04) 0.08 (0.01) 0.09 (0.00) | 0.32 (0.07) 0.35 (0.07) 0.14 (0.00) 0.08 (0.00) | - | 0.37 (0.07) 0.62 (0.04) 0.10 (0.01) 0.08 (0.01) |
| 6 | 5 | F1 Purity RMSEc RMSEt | 0.74 (0.09) 0.38 (0.03) 0.15 (0.01) 0.04 (0.01) | 0.60 (0.11) 0.43 (0.04) 0.10 (0.01) 0.05 (0.01) | 0.61 (0.10) 0.26 (0.02) 0.13 (0.01) 0.04 (0.01) | 0.64 (0.11) 0.39 (0.03) 0.12 (0.01) 0.04 (0.01) | 0.51 (0.12) 0.49 (0.04) 0.09 (0.01) 0.05 (0.01) | - | 0.28 (0.08) 0.31 (0.11) 0.10 (0.00) 0.05 (0.00) |
| 7 | 1 | F1 Purity RMSEc RMSEt | 0.25 (0.04) 0.56 (0.02) 0.21 (0.00) 0.21 (0.00) | 0.14 (0.04) 0.64 (0.03) 0.22 (0.00) 0.23 (0.00) | 0.20 (0.05) 0.73 (0.03) 0.21 (0.01) 0.21 (0.00) | 0.15 (0.04) 0.86 (0.04) 0.22 (0.00) 0.23 (0.00) | 0.23 (0.05) 0.85 (0.04) 0.21 (0.00) 0.21 (0.00) | - | - |
| 8 | 1 | F1 Purity RMSEc RMSEt | 0.28 (0.05) 0.55 (0.04) 0.19 (0.00) 0.18 (0.00) | 0.21 (0.05) 0.67 (0.04) 0.20 (0.00) 0.20 (0.00) | 0.15 (0.04) 0.79 (0.04) 0.20 (0.00) 0.21 (0.00) | 0.12 (0.04) 0.98 (0.01) 0.21 (0.00) 0.21 (0.00) | 0.13 (0.04) 0.87 (0.03) 0.22 (0.00) 0.21 (0.00) | - | 0.33 (0.05) 0.75 (0.06) 0.18 (0.01) 0.16 (0.01) |
| 8 | 2 | F1 Purity RMSEc RMSEt | 0.29 (0.05) 0.54 (0.03) 0.20 (0.00) 0.18 (0.00) | 0.18 (0.04) 0.63 (0.03) 0.19 (0.00) 0.20 (0.00) | 0.22 (0.05) 0.61 (0.04) 0.20 (0.01) 0.20 (0.00) | 0.17 (0.04) 0.68 (0.05) 0.21 (0.00) 0.21 (0.00) | 0.12 (0.04) 0.77 (0.06) 0.23 (0.00) 0.23 (0.00) | - | 0.29 (0.05) 0.83 (0.03) 0.20 (0.00) 0.21 (0.00) |
| 9 | 1 | F1 Purity RMSEc RMSEt | 0.55 (0.10) 0.24 (0.03) 0.19 (0.01) 0.05 (0.01) | 0.51 (0.15) 0.62 (0.13) 0.06 (0.00) 0.06 (0.00) | 0.55 (0.13) 0.60 (0.09) 0.07 (0.00) 0.06 (0.00) | 0.53 (0.13) 0.52 (0.08) 0.07 (0.00) 0.07 (0.00) | 0.46 (0.12) 0.70 (0.11) 0.06 (0.00) 0.06 (0.00) | - | 0.46 (0.11) 0.68 (0.11) 0.06 (0.00) 0.06 (0.00) |
| 9 | 2 | F1 Purity RMSEc RMSEt | 0.32 (0.05) 0.64 (0.03) 0.12 (0.01) 0.09 (0.01) | 0.17 (0.05) 0.70 (0.03) 0.15 (0.01) 0.15 (0.00) | 0.38 (0.05) 0.59 (0.02) 0.14 (0.01) 0.09 (0.01) | 0.27 (0.05) 0.72 (0.03) 0.10 (0.01) 0.10 (0.01) | 0.25 (0.05) 0.72 (0.02) 0.11 (0.01) 0.10 (0.01) | - | 0.36 (0.05) 0.45 (0.02) 0.21 (0.00) 0.09 (0.01) |
| 10 | 1 | F1 Purity RMSEc RMSEt | 0.33 (0.06) 0.58 (0.03) 0.23 (0.00) 0.25 (0.00) | 0.48 (0.06) 0.51 (0.02) 0.22 (0.00) 0.22 (0.01) | 0.31 (0.06) 0.64 (0.04) 0.24 (0.01) 0.25 (0.00) | 0.66 (0.05) 0.68 (0.02) 0.17 (0.01) 0.10 (0.01) | 0.67 (0.05) 0.55 (0.05) 0.25 (0.01) 0.13 (0.01) | - | 0.53 (0.05) 0.62 (0.12) 0.31 (0.01) 0.11 (0.01) |
| 10 | 2 | F1 Purity RMSEc RMSEt | 0.59 (0.06) 0.44 (0.02) 0.20 (0.01) 0.16 (0.01) | 0.26 (0.07) 0.71 (0.04) 0.19 (0.00) 0.19 (0.00) | 0.44 (0.07) 0.66 (0.02) 0.14 (0.01) 0.14 (0.01) | 0.30 (0.07) 0.72 (0.03) 0.19 (0.00) 0.19 (0.00) | 0.24 (0.07) 0.84 (0.03) 0.18 (0.01) 0.18 (0.01) | - | 0.33 (0.05) 0.66 (0.10) 0.24 (0.00) 0.13 (0.01) |
| 11 | 1 | F1 Purity RMSEc RMSEt | 0.42 (0.07) 0.18 (0.02) 0.18 (0.01) 0.06 (0.01) | 0.44 (0.15) 0.40 (0.05) 0.09 (0.01) 0.06 (0.00) | 0.45 (0.14) 0.24 (0.04) 0.11 (0.01) 0.06 (0.01) | 0.57 (0.13) 0.26 (0.03) 0.11 (0.00) 0.03 (0.01) | 0.51 (0.13) 0.23 (0.03) 0.11 (0.00) 0.04 (0.01) | - | 0.51 (0.13) 0.22 (0.02) 0.09 (0.00) 0.03 (0.01) |
| 11 | 2 | F1 Purity RMSEc RMSEt | 0.51 (0.11) 0.39 (0.05) 0.12 (0.00) 0.10 (0.01) | 0.57 (0.12) 0.45 (0.04) 0.10 (0.01) 0.08 (0.01) | 0.58 (0.11) 0.48 (0.04) 0.09 (0.01) 0.07 (0.01) | 0.77 (0.10) 0.43 (0.04) 0.13 (0.01) 0.04 (0.01) | 0.74 (0.10) 0.44 (0.03) 0.12 (0.01) 0.05 (0.01) | - | 0.70 (0.11) 0.40 (0.03) 0.12 (0.00) 0.04 (0.01) |
| 11 | 3 | F1 Purity RMSEc RMSEt | 0.67 (0.14) 0.48 (0.04) 0.08 (0.01) 0.05 (0.01) | 0.56 (0.14) 0.40 (0.07) 0.10 (0.00) 0.06 (0.01) | 0.59 (0.14) 0.36 (0.03) 0.11 (0.01) 0.06 (0.01) | 0.72 (0.11) 0.31 (0.03) 0.13 (0.00) 0.04 (0.01) | 0.71 (0.12) 0.35 (0.03) 0.12 (0.00) 0.04 (0.01) | - | 0.61 (0.12) 0.30 (0.03) 0.11 (0.00) 0.04 (0.01) |
| 12 | 1 | F1 Purity RMSEc RMSEt | 0.49 (0.09) 0.49 (0.05) 0.09 (0.01) 0.05 (0.01) | 0.43 (0.11) 0.53 (0.06) 0.08 (0.01) 0.06 (0.00) | 0.35 (0.12) 0.56 (0.06) 0.07 (0.01) 0.07 (0.01) | 0.43 (0.12) 0.57 (0.05) 0.06 (0.01) 0.05 (0.00) | 0.67 (0.11) 0.38 (0.04) 0.11 (0.01) 0.04 (0.01) | - | 0.69 (0.11) 0.39 (0.04) 0.10 (0.00) 0.04 (0.01) |
| 12 | 2 | F1 Purity RMSEc RMSEt | 0.52 (0.12) 0.37 (0.04) 0.12 (0.01) 0.06 (0.00) | 0.48 (0.13) 0.38 (0.05) 0.09 (0.01) 0.06 (0.00) | 0.46 (0.12) 0.42 (0.08) 0.09 (0.01) 0.06 (0.00) | 0.41 (0.15) 0.47 (0.06) 0.05 (0.01) 0.06 (0.01) | 0.47 (0.11) 0.26 (0.08) 0.09 (0.00) 0.04 (0.01) | - | 0.44 (0.11) 0.28 (0.08) 0.09 (0.00) 0.04 (0.01) |
| 12 | 3 | F1 Purity RMSEc RMSEt | 0.52 (0.11) 0.44 (0.05) 0.10 (0.01) 0.06 (0.01) | 0.60 (0.10) 0.39 (0.05) 0.13 (0.01) 0.06 (0.01) | 0.35 (0.10) 0.49 (0.08) 0.08 (0.01) 0.08 (0.01) | 0.30 (0.11) 0.33 (0.06) 0.09 (0.01) 0.08 (0.00) | 0.50 (0.10) 0.21 (0.02) 0.11 (0.00) 0.04 (0.01) | - | 0.37 (0.10) 0.17 (0.07) 0.10 (0.00) 0.07 (0.00) |
| 12 | 4 | F1 Purity RMSEc RMSEt | 0.52 (0.12) 0.42 (0.04) 0.11 (0.01) 0.08 (0.01) | 0.60 (0.10) 0.33 (0.06) 0.13 (0.01) 0.07 (0.01) | 0.35 (0.12) 0.65 (0.08) 0.07 (0.00) 0.07 (0.00) | 0.39 (0.11) 0.37 (0.04) 0.11 (0.01) 0.07 (0.00) | 0.41 (0.11) 0.21 (0.04) 0.12 (0.00) 0.07 (0.00) | - | 0.46 (0.11) 0.35 (0.03) 0.11 (0.00) 0.04 (0.01) |
| 12 | 5 | F1 Purity RMSEc RMSEt | 0.51 (0.10) 0.34 (0.03) 0.14 (0.01) 0.07 (0.01) | 0.47 (0.12) 0.52 (0.08) 0.07 (0.00) 0.07 (0.01) | 0.36 (0.14) 0.59 (0.08) 0.06 (0.01) 0.07 (0.00) | 0.47 (0.14) 0.48 (0.06) 0.06 (0.01) 0.06 (0.01) | 0.67 (0.13) 0.30 (0.04) 0.11 (0.00) 0.04 (0.01) | - | 0.58 (0.14) 0.29 (0.03) 0.10 (0.00) 0.04 (0.01) |

Table S4. Summary statistics from simulation 2.

|  |  | Simulation | | | | | | | | | | | |
| --- | --- | --- | --- | --- | --- | --- | --- | --- | --- | --- | --- | --- | --- |
|  | D | 1 | 2 | 3 | 4 | 5 | 6 | 7 | 8 | 9 | 10 | 11 | 12 |
| $\bar{\mathrm{Inertia}}$ | **0.003** | 0.03 | 0.035 | 0.022 | 0.018 | 0.024 | 0.022 | **0.009** | **0.009** | 0.025 | 0.044 | 0.024 | 0.022 |
| R^2^ | **0.008** | 0.060 | 0.087 | 0.033 | 0.066 | 0.067 | 0.179 | **0.012** | **0.021** | 0.032 | 0.139 | 0.061 | 0.098 |

Table S5. Comparison of the measured time-series effect size between David et al. (D) and the simulations (1-12) from simulation 2. Inertia is the mean constrained inertia from CCA with the intervention(s) as a covariate. R^2^ represents the variation explained by these covariates when performing PERMANOVA. Effect sizes closest to David et al. are shown in bold.

|  |  | Simulation | | | | | | | | | | | |
| --- | --- | --- | --- | --- | --- | --- | --- | --- | --- | --- | --- | --- | --- |
|  | D | 1 | 2 | 3 | 4 | 5 | 6 | 7 | 8 | 9 | 10 | 11 | 12 |
| $\bar{\mathrm{Inertia}}$ | **0.003** | 0.03 | 0.035 | 0.022 | 0.018 | 0.024 | 0.022 | **0.009** | **0.009** | 0.025 | 0.044 | 0.024 | 0.022 |
| R^2^ | **0.008** | 0.060 | 0.087 | 0.033 | 0.066 | 0.067 | 0.179 | **0.012** | **0.021** | 0.032 | 0.139 | 0.061 | 0.098 |

S2 Table. Comparison of the measured time-series effect size between David et al. (D) and the simulations (1-12) from simulation 2. Inertia is the mean constrained inertia from CCA with the intervention(s) as a covariate. R^2^ represents the variation explained by these covariates when performing PERMANOVA. Effect sizes closest to David et al. are shown in bold.
