# Supplementary material for: Exploring thematic structure and predicted functionality of 16S rRNA amplicon data
Source: PLoS One. 2019 Dec 11;14(12):e0219235. doi: 10.1371/journal.pone.0219235 (PMC6905537; doi:10.1371/journal.pone.0219235)
Supplement: S1 File — (DOCX) [file pone.0219235.s002.docx]

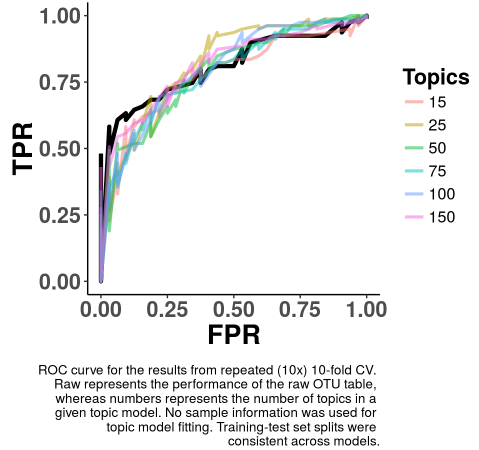


Figure A. ROC curve for Gevers data from repeated (10x) 10-fold CV with up-sampling, using an RF classifier. Performance of OTU relative abundances is shown in black, whereas numbers represent the number of topics in a given model. No sample information was used for topic model fitting. Training-testing set splits were consistent across models. TPR=True Positive Rate, FPR=False Positive Rate.


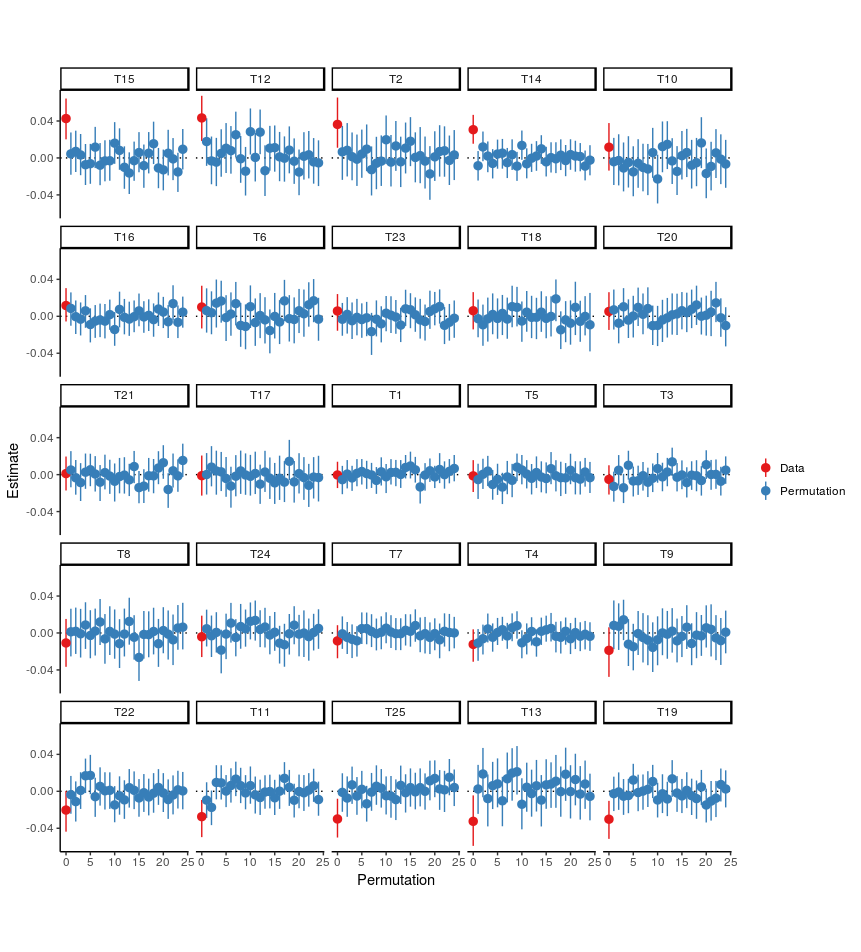
Figure B. Gevers estimates of the diagnosis covariate effect for K25 with prior information for true data (red) and 25 repetitions of class permutated data fits (blue). Shown are 95% uncertainty intervals. Facets are ordered by increasing mean for the true data fit (top-left to bottom-right).


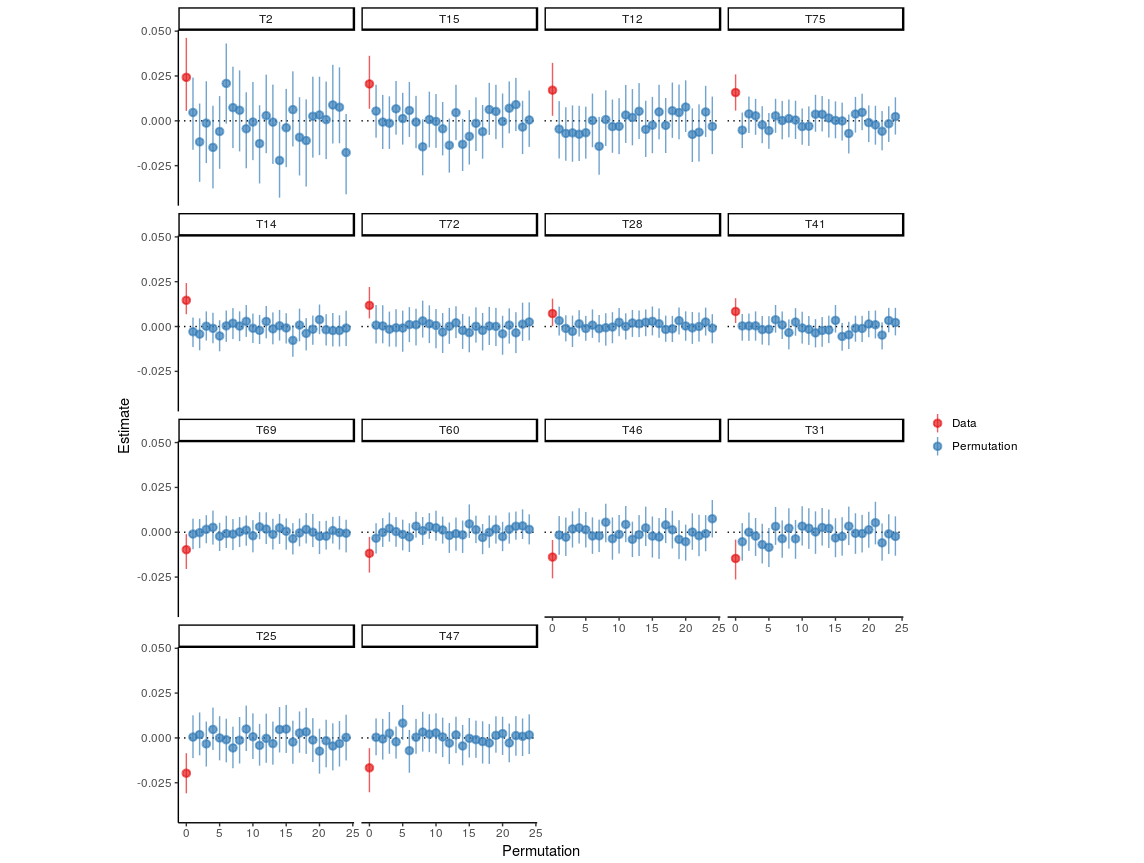


Figure C. Gevers estimates of the diagnosis covariate effect for K75 with prior information for true data (red) and 25 repetitions of class permutated data fits (blue). Shown are 95% uncertainty intervals. Facets are ordered by increasing mean for the true data fit (top-left to bottom-right). Topics with data estimates that spanned 0 are omitted from the figure due to size.


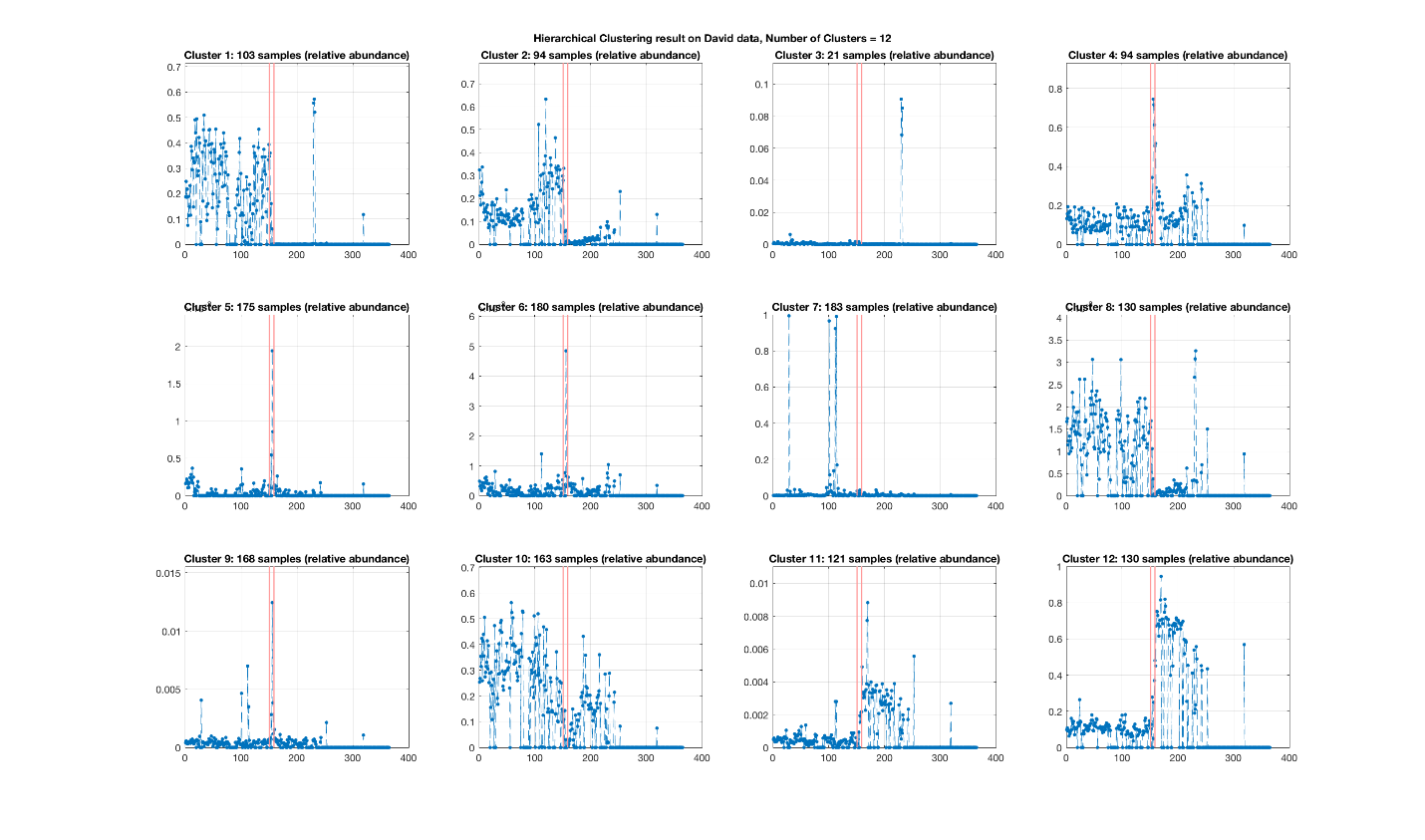


Figure D. Clusters via hierarchical clustering (k=12) applied to the David et al. dataset (subset B). Red lines signify the presentation of illness.

|  |  |  | Disease Status |  |
| --- | --- | --- | --- | --- |
|  | Samples | OTUs | CD+ | CD- |
| Total | 555 | 1500 | 375 | 180 |
| Training | 444 | 1500 | 296 | 148 |
| Testing | 111 | 1500 | 79 | 32 |

Table A. The distribution of Gevers samples and words across 80/20 training/testing splits, where CD+ and CD- represent the positive and negative diagnosis for CD, respectively.


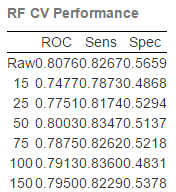


Table B. AUC, sensitivity, and specificity scores for Gevers data from repeated (10x) 10-fold CV with up-sampling, using an RF classifier. Performance of OTU relative abundances in designated as “Raw,” whereas numbers represent the number of topics in a given model. No sample information was used for topic model fitting. Training-testing set splits were consistent across models.


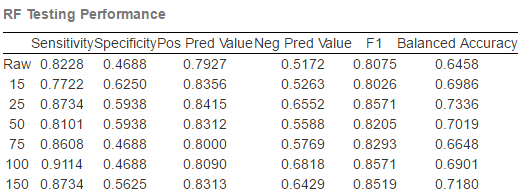


Table C. Generalization error for Gevers data during testing from repeated (10x) 10-fold CV with up-sampling, using an RF classifier. Performance of OTU relative abundances in designated as “Raw,” whereas numbers represent the number of topics in a given model. No sample information was used for topic model fitting. Training-testing set splits were consistent across models.


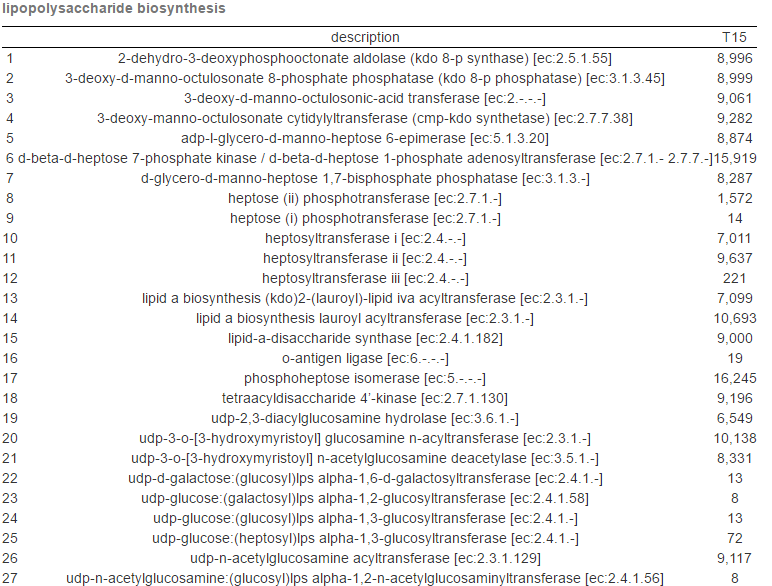


Table D. The predicted gene abundances in topic T15 for specific KOs that belonged to the level 3 KEGG category “lipopolysaccharide biosynthesis.” Pathway Category carbohydrate metabolism amino acid metabolism lipid metabolism cell motility glycan biosynthesis and metabolism xenobiotics biodegradation and metabolism metabolism of cofactors and vitamins nucleotide metabolism metabolism of terpenoids and polyketides biosynthesis of other secondary metabolites energy metabolism metabolism membrane transport cellular processes and signaling genetic information processing

| Pathway Category |
| --- |
| carbohydrate metabolism |
| amino acid metabolism |
| lipid metabolism |
| cell motility |
| glycan biosynthesis and metabolism |
| xenobiotics biodegradation and metabolism |
| metabolism of cofactors and vitamins |
| nucleotide metabolism |
| metabolism of terpenoids and polyketides |
| biosynthesis of other secondary metabolites |
| energy metabolism |
| metabolism |
| membrane transport |
| cellular processes and signaling |
| genetic information processing |

Table E. Level 2 KEGG pathway categories used in the multilevel negative binomial regression model. KOs not falling within these categories were removed. For level 3 analysis, only level 3 categories falling within this set of level 2 categories were used.


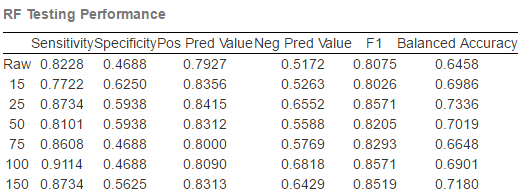


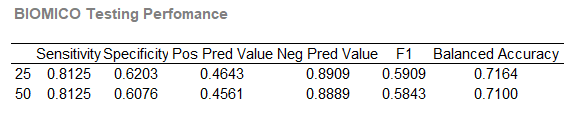


Table F. Generalization error for BioMico during testing using models trained with 25 and 50 assemblages.
